# Activity-dependent modulation of hippocampal synaptic plasticity via PirB and endocannabinoids

**DOI:** 10.1038/s41380-018-0034-4

**Published:** 2018-04-18

**Authors:** Maja Djurisic, Barbara K. Brott, Nay L. Saw, Mehrdad Shamloo, Carla J. Shatz

**Affiliations:** 1 Departments of Biology and Neurobiology, and Bio-X, Stanford University, Stanford, CA 94305 USA; 2 Behavioral and Functional Neuroscience Laboratory, Stanford University School of Medicine, Stanford, CA 94305 USA; 3 Behavioral and Functional Neuroscience Laboratory and Department of Neurosurgery, Stanford University School of Medicine, Stanford, CA 94305 USA

**Keywords:** Neuroscience, Physiology, Schizophrenia

## Abstract

The threshold for Hebbian synaptic plasticity in the CNS is modulated by prior synaptic activity. At adult CA3-CA1 synapses, endocannabinoids play a role in this process, but how activity engages and maintains this retrograde signaling system is not well understood. Here we show that conditional deletion of Paired Immunoglobulin-like receptor B (PirB) from pyramidal neurons in adult mouse hippocampus results in deficient LTD at CA3-CA1 synapses over a range of stimulation frequencies, accompanied by an increase in LTP. This finding can be fully explained by the disengagement of retrograde endocannabinoid signaling selectively at excitatory synapses. In the absence of PirB, the NMDAR-dependent regulation of endocannabinoid signaling is lost, while CB1R-dependent and group I mGluR-dependent regulation are intact. Moreover, mEPSC frequency in mutant CA1 pyramidal cells is elevated, consistent with a higher density of excitatory synapses and altered synapse pruning. Mice lacking PirB also perform better than WT in learning and memory tasks. These observations suggest that PirB is an integral part of an NMDA receptor-mediated synaptic mechanism that maintains bidirectional Hebbian plasticity and learning via activity-dependent endocannabinoid signaling.

## Introduction

Recent genome-wide association studies (GWAS) of Schizophrenia, now powered by impressive numbers of patients and controls, have identified the MHC locus (Major Histocompatibility Class I and II; Human HLA) as a major region of association [1, 2]. Single nucleotide polymorphisms associated with both classical and nonclassical MHC class I (MHCI) genes have been reported [1, 2]. In addition, MHCI gene expression and regulation by inflammation and nicotine are altered in the brains of patients with Schizophrenia [3]. Other genes at the HLA locus including components of the complement cascade, as well as MHC class II (HLA-DPA1 and HLA-DRB1), have been associated with schizophrenia, bipolar disorder and major depressive disorder [4–6]. Given their well-known roles in the immune system, these associations have been interpreted to imply that disorders of immune function may contribute to Schizophrenia.

Recently, however, it has become evident that particular MHCI proteins are expressed by neurons and are located at synapses in the healthy brain [7–9]. Also located in the MHC locus are components of the complement cascade, some of which are also expressed in neurons [4, 10]. Mice lacking C1q, C4 or the classical MHCI molecules H2-Db and H2-Kb all share remarkably similar synapse pruning deficits during an early developmental critical period in the visual system [4, 5, 11]. These observations point to a specific function of these molecules at synapses, as well as in the immune system, and suggest molecular mechanisms for the observed changes in synapse and spine density noted in the brains of Schizophrenia patients [12].

In the immune system, many MHCI family members act via cognate receptors, generating downstream signaling cascades. Paired Immunoglobulin-like receptor B (PirB; human LilrB2 and 3), an MHCI innate immune receptor [13], is expressed in excitatory pyramidal neurons of the forebrain [14]. In visual cortex of mice lacking PirB, the density of dendritic spines and functional glutamatergic synapses is more than 50% greater than WT [15]. This elevation in density persists into adulthood and matches closely the normal spine density measured at the onset of developmental synapse pruning [16], demonstrating a role for PirB in pruning [16]. It is not known how PirB regulates pruning, but it is possible to trigger the rapid appearance of new synapses in the brain of adult WT mice by acutely blocking PirB function using a recombinant decoy receptor [17]. Cellular mechanisms of synaptic weakening such as LTD are thought to be necessary for synapse pruning [11, 18, 19]. These mechanisms are best understood at CA3-CA1 hippocampal synapses [19, 20]. Here we examine LTP and LTD in mice with conditional deletions of PirB in hippocampal pyramidal neurons. A shift in the threshold for synaptic plasticity also prompted us to investigate endocannabinoid retrograde signaling, as well as behavior on simple memory tasks.

## Materials and methods

All experiments were carried out in accordance with the Guide for the Care and Use of Laboratory Animals of the National Institutes of Health and approved by the Stanford University Institutional Animal Care and Use Committee. Methods are also in accordance with the Policies of the Society for Neuroscience on the Use of Animals and Humans in Neuroscience Research. All mice were maintained in a pathogen-free environment. No statistical a priori sample size estimate was conducted. Behavioral and physiology studies of germline PirB−/− were all performed and analyzed blind to genotype. Experiments using different PirB fl/fl lines were analyzed blind to genotype, with the exception of an experiment on Pyr-WT vs. Pyr-KO for Fig S9 (C and D) which was both performed and analyzed blind to genotype. All experimental cohorts were block-randomized over time, and test litters were all age-matched and all male. The sampling quota was determined when statistical power was reached.

### Experimental model and subject details

Germline PirB−/− mutant mice, mice with postnatal conditional deletion of PirB, as well as corresponding WT cohorts, were obtained from a PirBfl/fl mouse line crossed with the appropriate Cre-deleter line. Generation of the PirBfl/fl line has been described previously [14], and detailed information is also provided in Supplementary Information (SI). All studies were performed in adult male animals or slices at postnatal day 90 (P90) or older.

Postnatal conditional deletion of PirB from forebrain pyramidal neurons was achieved by crossing PirBfl/fl to a line in which Cre recombinase is under the control of the CamKIIα promoter (B6.Cg-Tg(Camk2a-cre)T29-1Stl/J; JAX 005359). For CA3 specific Cre-mediated PirB deletion, the C57BL/6-Tg(Grik4-cre)G32-4Stl/J line was used (JAX 006474). Heterozygous mice (CamKII-Cre; PirB+/fl or Grik4-Cre; PirB+/fl) were bred to obtain litters containing both experimental and control genotypes (CamKIIa-Cre; PirBfl/fl (Pyr-KO) vs. CamKIIa-Cre; PirB+/+(Pyr-WT) or Grik4-Cre; PirBfl/fl (CA3-Pyr-KO) vs. Grik-4-Cre; PirB+/+ (CA3-Pyr-WT)). An automated genotyping service was used (Transnetyx, Inc.). Breeding strategy and genotyping considerations are provided in SI.

### Quantification and statistical analysis

Statistics for each experiment are reported in the accompanying Figure Legends. Data analysis and statistical analyses were performed using SigmaPlot 10.0 (Systat Software Inc.) and IBM SPSS Statistics 23 (IBM Corporation), respectively. Data are reported as mean ± s.e.m, with sample size given as number of slices or cells along with the number of mice (e.g., *n* = *x* slices/*y* mice); for behavioral experiments only the number of mice is reported. For behavioral and synaptic plasticity experiments, Two-way Anova with repeated measures (2-way RM Anova) was used to test differences between genotypes over time; the reported differences are due to effect of genotype, unless otherwise stated. One-way Anova was used to test differences between baseline and post-induction LTP/LTD period for a single genotype. One-Way Anova with post-hoc Tukey was used when needed to control for multiple comparisons. Data sets were comparable in variance (Levene’s test). The effect of plasticity-induction paradigms on fEPSP slopes and Paired Pulse Ratio (PPR) was assessed once a stable change was reached during the post-induction period. For synaptically evoked LTP/LTD, measurements were made starting at 20 min post-induction through the end of the recording period. For chemically induced LTD (e.g., with DHPG or NMDA), averages of fEPSP slopes or PPR were compiled starting at 30 or 40 min post-induction, as noted. Pairwise Mann–Whitney (U) test (normal distribution not assumed) was also used to compare means where appropriate. All statistical tests were two-tailed. Statistical significance was reached when probability due to chance fell below 5%; i.e., *p*-value *p* < 0.05. Outcomes of statistical tests were reported as exact p values, unless the probability due to chance fell below 0.1%, in which case it was always denoted as *p* < 0.001.

Detailed methods for delayed match to place behavioral test, hippocampal synaptic physiology, histology, and Western blotting are included in Supplementary Information.

## Results

### PirB null mice outperform WT on a delayed match-to-place task

**Fig. 1:**
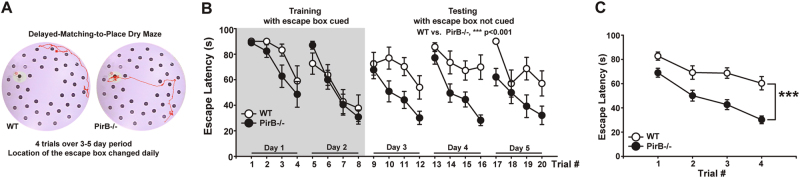
Faster learning in delayed-match-to-place (DMP) dry maze test in PirB−/− mice. **a** Photograph of modified Dry Barnes Maze Arena used for DMP task. Each mouse had four trials to find the escape box on each day, over a period of 5 days. Median tracks (red trace) from WT and PirB−/− at Day 4 /Trial 4 are superimposed on maze image; white patch indicates location of escape box. In this trial, WT never finds the box, while PirB−/− is successful. **b** Plot of escape latencies for 2 days of cued (Day 1, Day 2), followed by 3 days of non-cued (Days 3−5), trials. WT and PirB−/− mice performed the task equally well when a visual cue marked escape box during first two testing days (gray area on left); *p* = 0.204 for WT vs. PirB−/−, 2-way RM Anova. Escape latencies for non-cued trials over the next 3 day period shows steeper learning curves for PirB−/− than WT; performance is almost 3 × better for PirB−/− vs. WT on second day of non-cued trials; *p* < 0.001 across 3 days of non-cued testing; 2-way RM Anova. **c** Trials averaged across Day 3, 4, and 5 (non-cued testing) also show significantly better performance by PirB−/− vs. WT; ***p < 0.001; 2-way RM Anova. **b**, **c** WT (open symbols): *n* = 8 mice; PirB−/− (black symbols): *n* = 13 mice (See also Fig. S1)

As a first step in motivating a study of hippocampal synaptic plasticity in mice lacking PirB function (PirB−/−), hippocampal learning and memory were assessed in a delayed-match-to-place task (DMPT) using a modified Barnes dry maze (Fig. 1a) [21]. Intact LTP at hippocampal synapses, and functional NMDA receptors are thought to be needed for memory encoding during this task [22]. Proper execution of DMPT requires mice to form a stable long-term memory of spatial cues used for navigation. At the same time, as the position of an escape hole changes daily, animals have to acquire additional information that is used only for a period of time; this procedural aspect of DMPT is thought to reflect learning flexibility [22].

Both genotypes performed equally well during initial training (Fig. 1b). After the 2-day training period, mice were tested for an additional 3 days (Day 3 to Day 5) using the same visual cues placed around the arena, but now with an unmarked escape box, whose position changed daily. PirB−/− mice performed better than WT, with significantly shorter escape latencies across all trials for Day 3, 4, and 5 (Fig. 1b). Even on the first day (Day 3) of non-cued testing, escape latencies were significantly shorter in PirB−/−, with a 3 fold better performance achieved during the 4th trial on Day 4. A difference in escape latency is also evident when trials were averaged across 3 days of testing (Fig. 1c). Other measures such as the time and distance “savings” that mice achieve between the first and last trial of each day were also significantly larger in PirB−/− vs. WT (Fig. S1). Thus, PirB−/− mice successfully acquire a DMP task, and their performance exceeds that of WT, implying that learning capacity on this simple task is restricted by PirB.

It is possible that PirB deletion results in changes in basal synaptic transmission or intrinsic excitability of neurons involved in learning circuitry in hippocampus. Multiple parameters of CA3-CA1 synaptic function known to be implicated in learning and working memory [23–26] were measured, including I_AMPA_/I_NMDA_ ratio, kinetics of I_AMPA_ and I_NMDA_, PPF [27], as well as the firing rate and firing threshold of CA1 pyramidal cells (Fig. S2 A-E). Together, all these parameters of basal synaptic transmission and intrinsic excitability are intact in the absence of PirB. Nevertheless, input/output curves for CA3-CA1 synapses point to an increase in the strength of excitatory synaptic transmission in PirB−/−, relative to WT (Fig. S2F). This observation suggests that a perturbation in synaptic transmission such as the presence of more synapses and/or more powerful synapses may underlie the better performance of PirB−/− on DMPT.

### Conditional deletion of PirB in excitatory pyramidal cells abolishes LTD and lowers the threshold for LTP

Improved hippocampal-dependent learning in mice frequently correlates with larger LTP, and smaller LTD [28, 29]. To assess if Hebbian synaptic plasticity is altered in the absence of PirB, LTP and LTD were studied in acutely isolated hippocampal slices from adult mice (P90-P130). Prior work demonstrated that PirB expression in cerebral cortical pyramidal neurons is required for synaptic pruning [16]. Therefore, we used mice with conditional deletion of PirB only from pyramidal neurons [17] (Fig. S3). Conditional mice were generated by crossing PirBfl/fl mice with a CamKIIa-Cre deleter line (see SI). For simplicity, the abbreviated names of experimental and control lines used in these experiments are: 1) Pyr-KO for CamKIIa-Cre; PirB fl/fl, and 2) Pyr-WT for CamKIIa-Cre; PirB + / +.

**Fig. 2:**
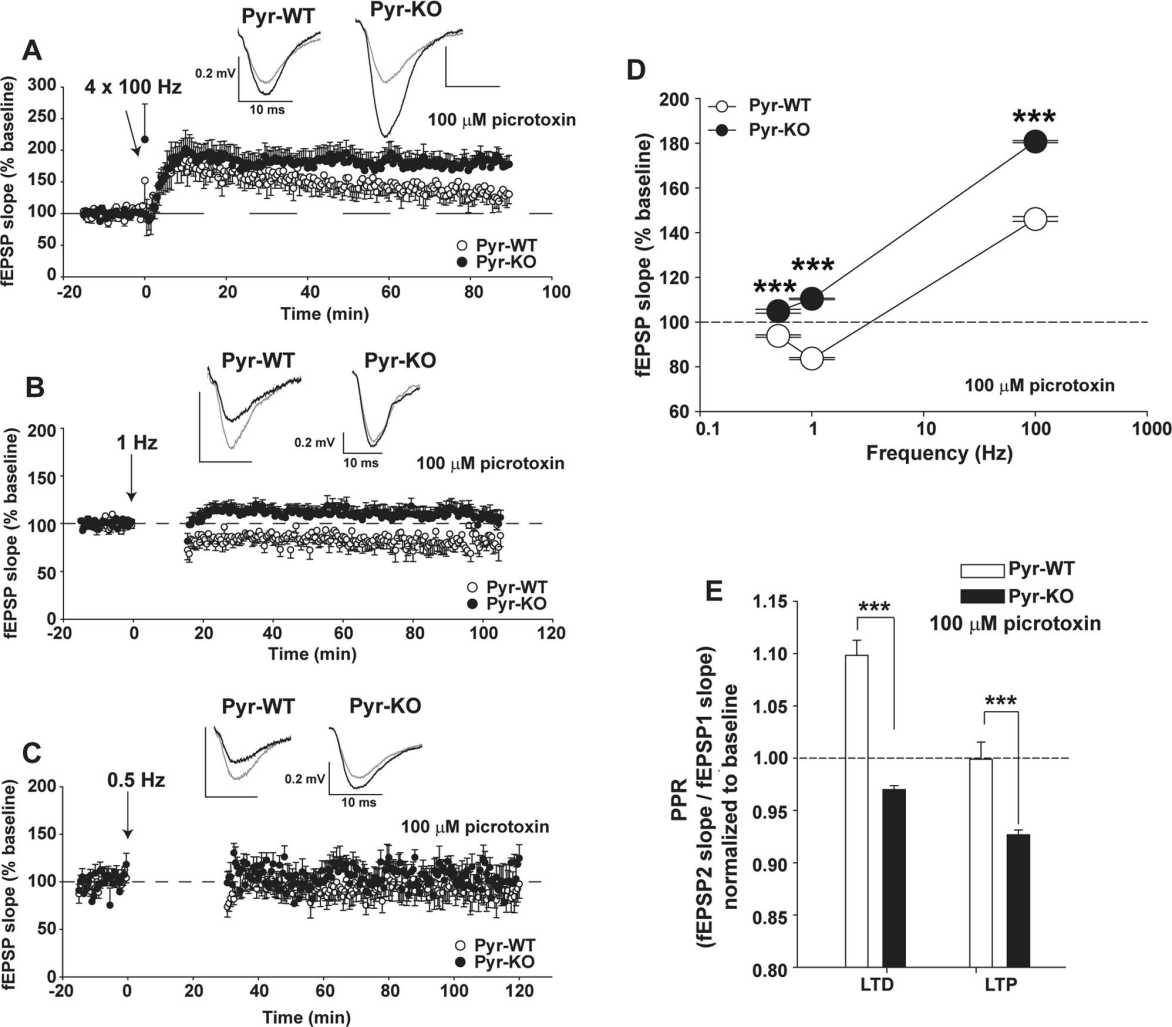
Deficient LTD and increased LTP with conditional deletion of PirB in forebrain excitatory neurons. **a** LTP at CA3-CA1 synapses in CamKIIa-Cre; PirB + / + (Pyr-WT) vs. CamKIIa-Cre; PirB fl/fl (Pyr-KO) induced with 4 × 100 Hz. Pyr-WT LTP: 146.19 ± 1.3 % (*n* = 8 slices/5 mice). Pyr-KO LTP: 180.7 ± 1.35 % (*n* = 8 slices/3 mice); *p* < 0.001. **b** LTD induced with 900 pulses at 1 Hz. Pyr-WT LTD: 83.9 ± 0.65 % (*n* = 12 slices/6 mice) vs. Pyr-KO LTD: 110.4 ± 0.61 % (*n* = 16 slices/6 mice); *p* < 0.001. **c** Same as (**b**), but LTD induction using 0.5 Hz. Pyr-WT LTD 0.5 Hz: 93.7 ± 1.15 % (*n* = 9 slices/5 mice) vs. Pyr-KO LTD 0.5 Hz: 104.8 ± 1.08 % (*n* = 5 slices/3 mice); *p* < 0.001. **d** Summary of LTP and LTD vs. the frequency of induction in Pyr-WT vs. Pyr-KO. Absence of LTD in Pyr-KO is evident for a range of frequencies from 0.5 to 400 Hz. **e** Bar-graphs of average paired-pulse ratios (PPR). Pyr-WT LTD: 1.10 ± 0.01 (*n* = 12 slices, 6 mice) vs. Pyr-KO LTD: 0.970 ± 0.003 (*n* = 16 slices, 6 mice); *p* < 0.001. Pyr-WT LTP: 0.999 ± 0.16 (*n* = 8 slices, 5 mice) vs. Pyr-KO LTP: 0.927 ± 0.004 (*n* = 8 slices, 3 mice), *p* < 0.001, ***p < 0.001. Test: 2-Way RM Anova. Mean values are averages of fEPSP slopes or PPR measured between 20–90 min post-induction. Insets**:** fEPSP trace examples from Pyr-WT and Pyr-KO slices before (gray line) and after induction (black line) of LTP (a) or LTD (b, c); each trace is average of 30 individual traces taken at baseline or 75–90 min post-induction.

The strength of CA3-CA1 synapses was measured with field potential recordings (Fig. 2). To assess activity-dependent strengthening at CA3-CA1 synapses, LTP was induced using 4 trains at 100 Hz. In Pyr-WT, a ~145% increase in fEPSP slope relative to baseline resulted. In adult Pyr-KO littermates, the same induction protocol generated significantly larger (~180%) and more persistent LTP (Fig. 2a). Next, LTD was induced in Pyr-KO or Pyr-WT littermates by delivering 900 pulses at 1 Hz. LTD was detected in Pyr-WT. In contrast, LTD was absent in Pyr-KO littermates; instead a small but significant LTP was observed (Fig. 2b).

Both LTP and LTD at CA3—CA1 synapses in WT animals are NMDA-receptor dependent [30, 31]. As expected, this dependence is seen in Pyr-WT: in the presence of the NMDA receptor blocker D-AP5, both LTP and LTD were abolished (Fig. S4). In Pyr-KO mice, LTP was also abolished with D-AP5, indicating that enhanced LTP is entirely dependent on NMDA receptors.

In the absence of PirB, LTD might still be present at CA3-CA1 synapses, but the threshold for induction could have shifted. To examine this possibility, LTD in Pyr-KO and Pyr-WT littermates was tested again, but at a frequency of 0.5 Hz. At this frequency, induction of LTD in Pyr-WT littermates is observed, but is about 91% of baseline—not as robust as at 1 Hz, and, similar to previous reports [32]. However, in Pyr-KO the same LTD induction protocol failed to generate LTD and instead resulted in a small, but significant (*p* < 0.001) potentiation—about 104% above baseline (Fig. 2c). Thus, frequency dependency has shifted away from LTD towards LTP in the absence of PirB (Fig. 2d). Together, these results suggest that PirB expression in excitatory neurons in adult hippocampus is needed for bidirectional changes in synaptic strength in an activity-dependent and NMDAR-dependent fashion.

### Activity-dependent modulation of paired pulse ratio is altered in Pyr-KO

Since changes in presynaptic function could account for observed alterations in LTP and LTD, paired-pulse ratio (PPR) was monitored continuously both at baseline and following induction of LTP or LTD (Fig. 2e; Fig. S5). PPR is thought to reflect transmitter release, with an increase in PPR associated with lowered glutamate release and vice versa [27, 33]. As expected from previous studies, PPR in Pyr-WT increases following LTD induction [34]. This activity-dependent modulation of PPR is consistent with lower glutamate release [27]. In contrast, in Pyr-KO the same LTD protocol resulted in an unexpected decrease in PPR (Fig. 2e). For LTP, following induction in Pyr-WT, there is no change in PPR, again as expected [35]. In contrast in Pyr-KO, PPR decreased by about 7 % (Fig. 2e) after LTP induction. It is worth noting that baseline PPR, prior to LTP or LTD induction, is comparable between Pyr-WT and Pyr-KO mice (Fig. S5C), as is between germline PirB−/− vs. WT (Fig. S2D), and thus baseline differences between Pyr-WT and Pyr-KO do not contribute to the change in PPR observed in the post-induction period. In sum, in Pyr-KO there is a paradoxical decrease in PPR following both LTP, as well as LTD (Fig. 2e), suggesting that PirB may contribute to activity-dependent regulation of glutamate release at CA3-CA1 synapses.

### Blockade of endocannabinoid receptor CB1R in Pyr-WT phenocopies Pyr-KO LTD and LTP

At CA3-CA1 synapses, presynaptic release is modulated by retrograde effects of endocannabinoids (eCBs) in an activity-dependent way: eCBs are produced and released from CA1 dendrites using LTP-inducing paradigms (100 Hz tetanus or theta burst), and are also critical for induction of both I-LTD throughout the CA1 dendritic tree [36–39] and for LTP at excitatory CA3-CA1 synapses [40]. LTD requires postsynaptic release of eCBs, which in turn decreases glutamate release presynaptically [34, 41].

**Fig. 3:**
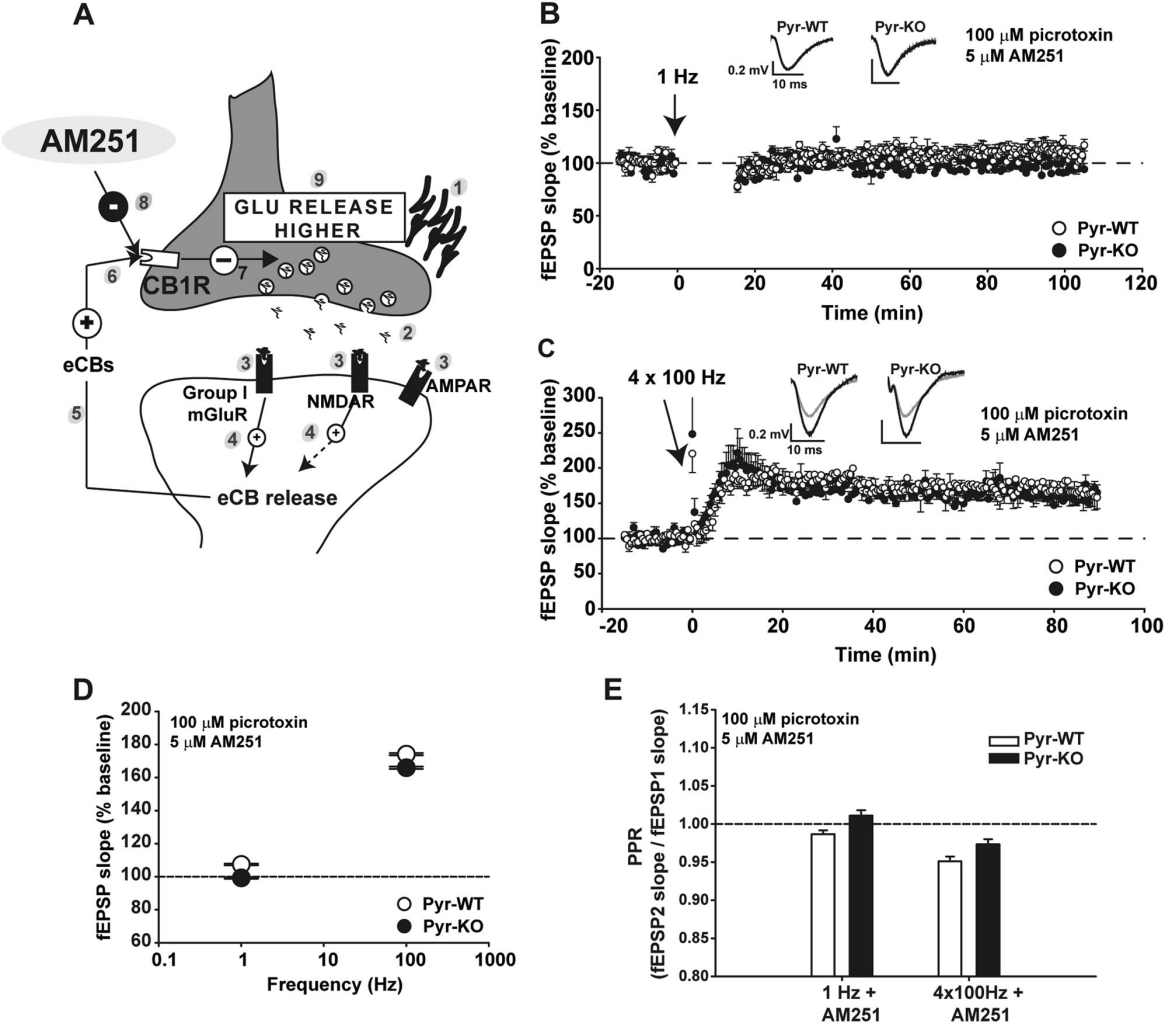
Blockade of CB1R in Pyr-WT phenocopies LTP and LTD alterations in Pyr-KO. **a** Cartoon illustrating synthesis and retrograde release of eCBs following a plasticity-inducing stimulus. Synaptic stimulation [1] triggers release of glutamate [2] which binds to postsynaptic AMPAR, NMDAR and Group I mGluRs [3]. Signaling downstream of NMDAR and Group I mGluRs elicits endogenous cannabinoid synthesis (eCBs) and release [4, 5], and binding to presynaptic CB1 receptor (CB1R) [6]. CB1R activation decreases neurotransmitter release [7]. Application of AM251 (a CB1R inverse agonist) depresses CB1R activation [8] and generates higher than normal glutamate release [9]. **b** LTD (1 Hz) of CA3-CA1 synapses is abolished in Pyr-WT with addition of 5 µM AM251 (107.4 ± 0.57 % baseline; *n* = 10 slices/4 mice); 1 Hz stimulation in Pyr-KO in the presence of AM251 resulted in the post-induction fEPSP slope of 99.2 ± 0.63 % baseline (*n* = 8 slices/4 mice). **c** LTP in Pyr-WT in the presence of 5 µM AM251 (174.1 ± 1.2 %; *n* = 13 slices/5 mice) increases relative to LTP in control conditions (146.19 ± 1.3 % from Fig. 2a, *p* < 0.001, 2-Way RM Anova), and even slightly surpasses the LTP measured in Pyr-KO in the presence of 5 µM AM251 (165.8 ± 1.5%; *n* = 8 slices/3 mice; *p* < 0.001, 2-Way RM Anova). **d** Summary of LTP and LTD vs. frequency of induction in Pyr-WT vs. Pyr-KO in presence of 5 µM AM251. Absence of LTD is now evident in both Pyr-KO and Pyr-WT. **e** PPR in AM251. Differences in PPR in Pyr-WT vs. Pyr-KO after induction of LTD and LTP seen in control conditions (Fig. 2e) are abolished in the presence of AM251.PPR after 1 Hz: Pyr-WT 0.99 ± 0.01 (n = 10 slices/ 4 mice) vs. Pyr-KO, 1.01 ± 0.01 (*n* = 8 slices/ 4 mice); *p* = 0.02. PPR after 4 × 100 Hz: Pyr-WT: 0.95 ± 0.01 (*n* = 13 slices, 5 mice) vs. Pyr-KO: 0.97 ± 0.01 (*n* = 8 slices, 3 mice); *p* = 0.015. Test: 2-Way RM Anova. Means are averages from 20 to 90 min post-induction. Insets: fEPSP trace examples from Pyr-WT and Pyr-KO slices before (gray line) or after induction (black line) of LTD (b) or LTP (c); each trace is average of 30 individual traces taken at baseline or at 75–90 min post-induction.

To examine if an eCB-dependent component of plasticity is altered in Pyr-KO, LTD and LTP experiments were repeated in the presence of 5 µM AM251, an inverse agonist of CB1R, an endocannabinoid-specific receptor expressed in hippocampus (Fig. 3a) [42]. In Pyr-WT, blockade of CB1R abolished LTD, while LTP increased in size to match that of Pyr-KO. In Pyr-KO, the effect of AM251 was occluded (Fig. 3b, c, d). In addition, the difference in PPR between Pyr-WT and Pyr-KO observed during LTP and LTD in control conditions (Fig. 2e) was abolished with CB1R blockade (Fig. 3e). Together, these results suggest that in Pyr-KO, eCB release and CB1R signaling are disengaged, pointing to a role for PirB in eCB signaling at CA3-CA1 synapses during both LTD and LTP.

### Group I mGluR and CB1R signaling are intact in Pyr-KO

**Fig. 4:**
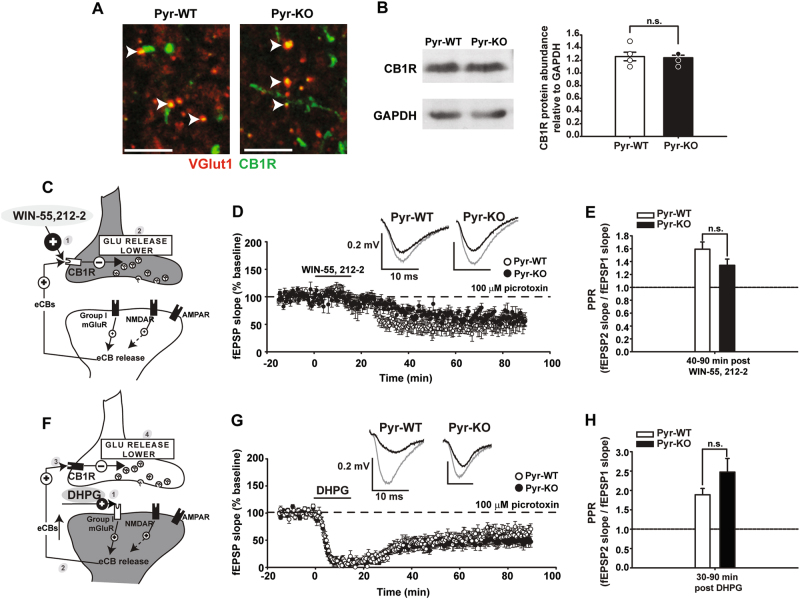
Activation of CB1R and group I mGluR, obligatory components of endocannabinoid system in CA3-CA1, is similar between Pyr-WT and Pyr-KO. **a** Fluorescent micrographs of CA1 *St. radiatum* from Pyr-WT (left) and Pyr-KO (right) showing VGlut1 (red) and CB1R immunostaining (green). Overlap of the two signals is yellow. No apparent difference is detectable between the two genotypes. Calibration bar: 5 µm. **b** Western Blot analysis for CB1R, relative to GAPDH, for Pyr-WT and Pyr-KO. Left—image of a representative gel showing CB1R specific and GAPDH specific bands. Right—bar graph of relative CB1R protein abundance in Pyr-WT (1.26 ± 0.07, *n* = 5) vs. Pyr-KO (1.24 ± 0.04, *n* = 5), *p* = 1.000, *U*-test. **c** Cartoon of CA3-CA1 synapse depicting effect of CB1R agonist WIN-55,212-2 [1] on CB1R activation, resulting in lower glutamate release [2]. **d** 15 min bath application of 5 µm WIN-55,212-2 generates persistent WIN-LTD in Pyr-WT (49.4 ± 0.95 %; *n* = 8 slices/4 mice; *p* < 0.001 relative to baseline) and Pyr-KO (65.9 ± 0.89 %; *n* = 7 slices/3 mice; *p* < 0.001 relative to baseline). **e** Average PPR for WIN-LTD in Pyr-WT: 1.59 ± 0.11 (*n* = 8 slices/4 mice) vs. Pyr-KO: 1.34 ± 0.10 (*n* = 7 slices/3 mice); *p* = 0.10. **f** Cartoon depicting effect of Group I mGluR agonist DHPG [1] on eCB release [2], eCB binding to CB1R [3] and lower glutamate release [4]. **g** 15 min bath application of 100 µm DHPG resulted in prominent DHPG-LTD in both Pyr-WT (53.25 ± 0.8 %; *n* = 11 slices/ 4 mice; *p* < 0.001 relative to baseline; one-way Anova) and Pyr-KO (45.1 ± 0.80%; *n* = 11 slices/ 4 mice; *p* < 0.001 relative to baseline; one-way Anova). **h** Average PPR after DHPG application. During DHPG-LTD (30–90 min post DHPG), PPR in Pyr-WT: 1.89 ± 0.16 (*n* = 11 slices, 4 mice) vs. PPR in Pyr-KO: 2.47 ± 0.35 (*n* = 11 slices, 4 mice); *p* = 0.621. 2-Way RM Anova. Insets**:** fEPSP trace examples from Pyr-WT and Pyr-KO slices before (gray line) and after induction (black line) of WIN-LTD (d) or DHPG-LTD (g); each trace is average of 30 individual traces taken at baseline or at 75–90 min post-induction.

Changes in abundance or function of CB1Rs in Pyr-KO hippocampus could explain why hippocampal synaptic plasticity in PirB mutant mice phenocopies that in WT mice when CB1 receptors are blocked. We confirmed the presence of presynaptically-located CB1Rs in glutamatergic terminals [43, 44] in P90 hippocampus—an age when CB1 receptors and eCB signaling are mature [45, 46]. In high resolution immunofluorescence images, using an antibody raised against CB1R combined with immunostaining for VGlut1 (a presynaptic marker of excitatory synapses; Fig. 4a), we observed that CB1R is present at a subset of CA3 boutons, as previously described [43]. In addition, CB1R immunostaining is associated with axonal varicosities and puncta, and is particularly abundant in CA1 *st. pyramidale*, as expected from its well-known expression on GABAergic presynaptic terminals (Fig. S6) [43]. Western Blot analysis indicates that CB1R protein levels are comparable in Pyr-KO vs. Pyr-WT (Fig. 4b). Thus, CB1R expression or protein localization is not significantly altered in the absence of PirB.

To test if functional activation of CB1R is altered in Pyr-KO, a chemical LTD paradigm was used. Activation of CB1R with bath application of the high-affinity agonist WIN-55,212-2 is known to generate LTD at CA3-CA1 synapses (Fig. 4c, d) due entirely to a decrease in glutamate release and is thus purely presynaptic [34, 47, 48]. WIN-55,212-2 generates chemical LTD (WIN-LTD) in both Pyr-KO and Pyr-WT mice to a similar degree (Fig. 4d). PPR is also increased in both genotypes after WIN application, as expected (Fig. 4e). These observations suggest that CB1R receptors are fully functional in Pyr-KO, and that signaling downstream of CB1R is also intact. Finally, the presence of intact WIN-evoked chemical LTD in Pyr-KO suggests that eCB signaling during synaptically-induced LTD is disrupted prior to the engagement of CB1Rs.

Group I mGluRs and NMDA receptors act upstream of CB1Rs, and each glutamate receptor subtype is known to trigger postsynaptic eCB release from CA1 neurons [34, 41, 49]. Group I metabotropic glutamate receptors (mGluRs) located postsynaptically are also thought to be recruited during synaptically-induced LTD under certain conditions (e.g., [50]). To test for PirB involvement in group I mGluR function, a chemical LTD paradigm was used again (Fig. 4f, g, h). Group I mGluR activation with the high-affinity agonist DHPG generates sustained DHPG-LTD at CA3-CA1 synapses in Pyr-WT. In Pyr-KO, application of DHPG had similar effects. In addition, PPR increased about 2.5 fold during DHPG-LTD in both genotypes [51]. To test if group I mGluRs, or downstream signaling components, are also recruited during 1 Hz synaptically-induced LTD [34, 41], DHPG-LTD was followed by 1 Hz synaptic stimulation (Fig. S8 A, B): 1 Hz induction failed to generate additional LTD in either Pyr-WT or Pyr-KO, indicating that downstream signaling common to activation of group I mGluRs, as well as synaptically-induced LTD is intact in Pyr-KO. PPR remained elevated following 1 Hz stimulation in both genotypes, implying that mGluR-dependent retrograde regulation of presynaptic release is intact.

### DSI is intact at inhibitory synapses on CA1 pyramidal cells

To assess if there might be changes in eCB release at other synapses onto CA1 pyramidal neurons in Pyr-KO, we examined a well-described eCB-dependent change in short-term plasticity at GABAergic synapses: depolarization induced suppression of inhibition (DSI) [52]. DSI is a consequence of the retrograde effect of depolarization-induced eCB release from CA1 pyramidal cells on presynaptic GABAergic terminals, leading to a decrease in the amplitude of IPSCs due to less GABA release [52]. Remarkably, DSI is intact in Pyr-KO, relative to Pyr-WT (Fig. S7). Thus, the retrograde effects of eCB release appear to be unaltered at inhibitory, but not excitatory, synapses on to CA1 pyramidal neurons, pointing to a restricted role of PirB at excitatory CA3 to CA1 synapses. Collectively these experiments demonstrate that CB1R function, activation of group I mGluRs, and effects of eCBs at GABAergic synapses are not affected in the absence of PirB. Moreover, they confirm a presynaptic location for CB1 receptors at glutamatergic CA3-CA1 Schaffer collateral synapses [34, 41, 43, 47].

### Chemical NMDA-dependent LTD is abolished in Pyr-KO

NMDA receptors are required for induction and maintenance of LTP and LTD at excitatory synapses, including CA3-CA1 [30, 31]. Activation of NMDARs is also known to contribute to the production of eCBs in apical dendrites of CA1 neurons [49]. If PirB is part of an NMDA receptor-dependent mechanism for eCB release, then there should be a change in NMDA-dependent synaptic plasticity in the absence of PirB.

**Fig. 5:**
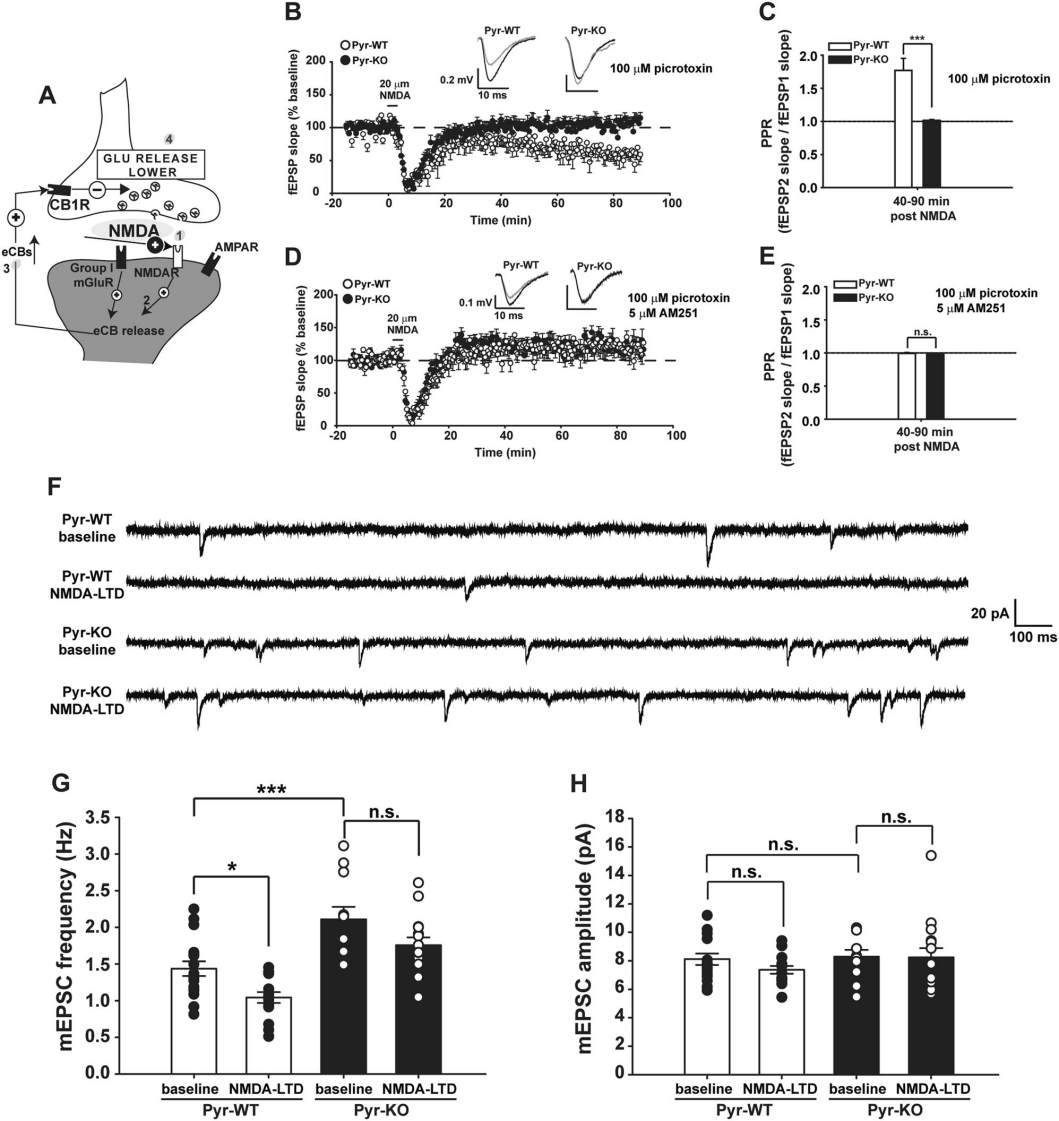
NMDA-LTD and NMDA-dependent synaptic pruning is abolished in Pyr-KO mice. **a** Cartoon of a CA3-CA1 synapse depicting the sequential effect of bath-application of NMDA [1] on eCB release [2], activation of CB1Rs [3], and lowering glutamate release [4]. **b** Three minute 20 µM NMDA induces persistent LTD in Pyr-WT (71.3 ± 0.96%; *n* = 12 slices/4 mice; *p* < 0.001 relative to baseline); no effect in Pyr-KO (103.6 ± 1.03 %; *n* = 10 slices, 4 mice); *p* < 0.001, 2-Way RM Anova. **c** PPR for NMDA-LTD in Pyr-WT: 1.76 ± 0.14 (*n* = 12 slices/4 mice) vs. Pyr-KO: 1.01 ± 0.16 (*n* = 10 slices, 4 mice); *p* < 0.001, 2-Way RM Anova. **d**  NMDA-LTD in Pyr-WT in the presence of 5 µM AM251: 120.3 ± 1.5 % (*n* = 11 slices/4 mice) vs. Pyr-KO: 122.3 ± 1.0 % (*n* = 8 slices/3 mice); *p* = 0.37, 2-Way RM Anova. **e**  PPR during NMDA-LTD in AM251 is comparable between Pyr-WT (0.994 ± 0.013; *n* = 11 slices/4 mice) vs. Pyr-KO (0.99 ± 0.015; *n* = 8 slices/3 mice); *p* = 0.73, 2-Way RM Anova. Data averaged during period from 40 to 90 min post-induction. **f** Example traces of mEPSCs from Pyr-WT and Pyr-KO before and after induction of NMDA-LTD with a brief (3 min) pulse of 20 µM NMDA. **g** Bar-graph of average mEPSC frequency, with overlaid individual cell values (black and white symbols). Pyr-WT baseline: 1.44 ± 0.10 Hz (*n* = 17 cells/6 mice) vs. Pyr-WT NMDA-LTD: 1.04 ± 0.07 Hz (*n* = 15 cells/6 mice); *p* = 0.047. Pyr-WT baseline vs. Pyr-KO baseline: 2.11 ± 0.17 Hz (*n* = 11 cells/4 mice); *p* = 0.001. Pyr-KO baseline vs. Pyr-KO NMDA-LTD: 1.76 ± 0.11 Hz (*n* = 15 cells/4 mice); *p* = 0.153. One-Way Anova with post-hoc Tukey for multiple comparisons. **h** Bar-graph of average mEPSC amplitude, with overlaid individual cell values (black and white symbols). Pyr-WT baseline: 8.11 ± 0.41 (*n* = 17 cells/6 mice) vs. Pyr-WT NMDA-LTD: 7.36 ± 0.27 (*n* = 15 cells/6 mice); *p* = 0.652; Pyr-KO baseline: 8.29 ± 0.48 (*n* = 11 cells/4 mice) vs. Pyr-KO NMDA-LTD: 8.23 ± 0.66 (*n* = 15 cells/4 mice); *p* = 1.00. Pyr-WT baseline vs. Pyr-KO baseline: p = 0.99. One-Way Anova with post-hoc Tukey for multiple comparisons.

To isolate NMDA-dependent mechanisms, a chemical NMDA-LTD paradigm was used (Fig. 5a). In Pyr-WT, brief application of 20 µM NMDA generates LTD at CA3-CA1 synapses (Fig. 5b), as expected [53, 54]). In contrast, in Pyr-KO, NMDA fails to produce sustained LTD (Fig. 5d). This result agrees well with our observation above that synaptically induced LTD in Pyr-KO is defective (Fig. 2), as well as with numerous published experimental results showing that blockade of NMDARs with MK801 or D-AP5 abolishes LTD [30, 31]. In Pyr-WT mice, PPR was increased (Fig. 5c), suggesting that bath-applied NMDA also decreases glutamate release, as expected if endocannabinoid release and retrograde signaling are NMDA-dependent [48, 49]. In Pyr-KO, PPR is unchanged with NMDA application (Fig. 5e), consistent with deficient LTD in these mice.

These differences between Pyr-KO and Pyr-WT are not due to changes in NMDA receptor abundance or function in Pyr-KO. Total protein levels of NR1, an obligatory NMDAR subunit, as well as NR2A and NR2B subunits, is comparable between Pyr-WT and Pyr-KO (Fig. S9A, B). Synaptic distribution of functional NMDARs, as assessed by measuring I_AMPA_/I_NMDA_ ratio was not changed in Pyr-KO vs. Pyr-WT (Fig. S9C). In addition, the kinetics of I_NMDA_ decay currents were comparable between the genotypes (Fig S9D), suggesting similar composition of NR2A and NR2B subunits in Pyr-KO NMDARs, relative to Pyr-WT. Finally, blockade of I_NMDA_ with bath-application of a selective antagonist of NR2B- containing NMDARs, Ro 25–6981, corroborated the conclusion that NR2A/2B composition of NMDARs in Pyr-KO is similar to Pyr-WT (Fig. S9E).

To confirm that eCB signaling can be engaged in NMDA-LTD in Pyr-WT mice, in a subset of experiments CB1Rs were blocked with AM251. As expected, blockade of CB1Rs prevented maintenance of NMDA-LTD in Pyr-WT, without an additional effect in Pyr-KO slices (Fig. 5d). The difference in PPR between the two genotypes observed during NMDA-LTD was also abolished in the presence of AM251 (Fig. 5e). Together, these observations strongly argue that without PirB, the NMDAR-dependent regulation of LTD via endocannabinoid signaling is abrogated.

### Synapse pruning driven by NMDA-dependent LTD is deficient in Pyr-KO

It is well established that LTD, either synaptically driven or chemically induced by NMDA, results in synaptic pruning, as evidenced by direct observation of dendritic spine shrinkage and removal in an NMDA-dependent manner within an hour of LTD induction [18, 19, 55, 56]. Elimination of functional synapses can also be monitored electrophysiologically, signaled by a decrease in the frequency of mEPSCs [57]. To examine if PirB expression in CA1 neurons is needed for LTD-induced synaptic pruning, mEPSCs were monitored in Pyr-WT vs. Pyr-KO slices before and then 60 min after induction of NMDA-LTD (Fig. 5f–h). In Pyr-WT, a 3 min pulse of NMDA generated a 30% decrease in mEPSC frequency (Fig. 5f, g), similar to previous reports [57]. In contrast, in Pyr-KO mEPSC frequency at baseline was already higher than that of Pyr-WT by about 50%, and remained unchanged even after NMDA application (Fig. 5f, g). No changes were observed in the amplitude of mEPSCs following NMDA application in either genotype (Fig. 5h). Thus, postnatal deletion of PirB specifically from pyramidal neurons renders synapses resistant to activity-dependent weakening and pruning in the context of acute changes elicited by NMDA-LTD. Moreover, the presence of increased mEPSC frequency in Pyr-KO over Pyr-WT at baseline suggests that there are already more functional excitatory synapses on CA1 neurons, which could explain stronger baseline excitatory transmission (Fig. S2F), and could also contribute to better performance on DMTP (Fig. 1).

### CA3-specific deletion of PirB preserves NMDA-LTD

Results suggest a model in which PirB is required postsynaptically within CA1 cells for an NMDA receptor-dependent eCB regulation of synaptic plasticity, and for the capacity of synapses to generate LTD (Figs. 2–5f). To further address the question of pre- vs. postsynaptic site of action of PirB in adult hippocampal plasticity, the NMDA-LTD plasticity paradigm was repeated, but this time in a mouse line in which PirB has been deleted only from CA3; this is in contrast to Pyr-KO mice, where PirB was excised both from CA3 and CA1. A line carrying a Cre-transgene driven by a CA3-specific promoter (Grik4-Cre; see SI) was crossed to PirBfl/fl mice to generate offspring carrying a deletion of PirB in CA3, but not CA1 pyramidal neurons (Fig. S10 A, B, C). These mice are henceforward referred to as CA3-Pyr-KO, and their WT littermates as CA3-Pyr-WT.

In CA3-Pyr-WT mice, a pulse of 20 µm NMDA generates chemical LTD at CA3-CA1 synapses (about 50% decrease from baseline) (Fig. S10D), similar to that seen in Pyr-WT (cf. Fig. 5b). In CA3-Pyr-KO, LTD indistinguishable from that seen in CA3-Pyr-WT is generated (Fig. S10D). Furthermore, in both CA3-Pyr-WT and CA3-Pyr-KO, there is a similar increase in PPR following NMDA application, consistent with the expression of LTD in both lines (Fig. S10E). Thus, when PirB deletion is restricted presynaptically to CA3 neurons, NMDA-LTD is intact. PirB might still be present in other neurons, even after deletion of PirB in CA3 or CA1 pyramidal cells, and thus somehow affect CA3-CA1 plasticity indirectly. However, in hippocampus these “other” neurons consist of inhibitory GABAergic interneurons. Because all plasticity measurements were conducted in the presence of 100 µM picrotoxin, and because PirB expression in GABAergic interneurons has not been detected, it is highly unlikely that other cell types contribute to the results described here. The conclusion that postsynaptically localized PirB is needed for NMDAR-dependent plasticity at CA3-CA1 synapses is also consistent with previous observations showing that mosaic deletion of PirB in isolated layer 2/3 pyramidal neurons of visual cortex is sufficient to generate increased spine density on apical and basal dendrites, implying a postsynaptic, cell-autonomous function of PirB [16]. Taken together, these observations suggest that PirB expressed postsynaptically in CA1 pyramidal cells, rather than presynaptically in CA3, is required for NMDAR-dependent LTD, and by extension, for NMDAR-dependent regulation of eCB signaling and Hebbian synaptic plasticity.

## Discussion

The major finding of this study is that PirB is required at excitatory CA3-CA1 synapses for activity-dependent recruitment of retrograde endocannabinoid signaling, a negative feedback system that acts to limit release of neurotransmitter. In mice lacking PirB, activity-dependent modulation of synaptic transmission is altered in such a way as to phenocopy wild type CA3-CA1 synapses in which endocannabinoid receptors (CB1R) have been blocked. While it is well known that eCBs act at GABAergic synapses in hippocampus in I-LTD [37], and in DSI [52] as we have confirmed above, previous studies have also demonstrated that eCBs are required for synaptically-induced LTD at CA3-CA1 excitatory synapses [34, 41]. Genetic removal or pharmacological blockade of CB1Rs renders these synapses unable to generate LTD in WT [34, 41], while LTP-inducing stimuli generate enhanced LTP [40]. These changes are exactly what were observed at CA3-CA1 synapses of mice with conditional deletion of PirB: LTD is absent across a range of plasticity-inducing stimuli from 0.5 Hz to 100 Hz (Fig. 2), and in fact only LTP of different magnitudes depending on stimulation parameters is found. Results also suggest that PirB and eCBs are part of the same pathway, because in hippocampus of mice lacking PirB the effect of blocking CB1Rs on both LTP and LTD is occluded. Together, these observations suggest that PirB is required for LTD, and is part of a synaptic system for regulating bidirectional Hebbian synaptic plasticity at excitatory synapses.

### A role for PirB in NMDAR, but not group I mGluR-dependent endocannabinoid signaling

It is remarkable that in the absence of PirB, only the NMDAR-dependent regulation of eCBs is deficient, while the group I mGluR dependent eCB pathway appears to be intact. Direct activation of Group I mGluRs with DHPG generates a similar magnitude of chemical LTD in both mutant and WT mice. However, when chemical LTD is induced by bath-applying NMDA, a high-affinity agonist of NMDA receptors, LTD was generated only in WT hippocampus, but was absent in mice lacking PirB. This observation suggests that some patterns of synaptic activity can engage eCB signaling downstream of NMDARs independently of group I mGluRs since one is PirB-dependent and the other is not. Previous studies have demonstrated the involvement of Ca^2+^ influx through NMDA receptors in triggering eCB release, as well as an eCB contribution to synaptically induced LTD at this synapse [48, 49, 58]. Our observations are entirely consistent with these studies, and also add a new molecular component to this signaling pathway: PirB.

The fact that hippocampal mGluR-dependent LTD is intact when PirB is removed from pyramidal cells argues that most if not all other postsynaptic and presynaptic components of eCB signaling are functional and independent of PirB. Whether endocannabinoid synthesis and release at CA3 to CA1 synapses is triggered by Group I mGluRs, voltage-gated calcium channels or NMDARs [34, 41, 48, 49], once released, eCBs bind to the endocannabinoid specific receptor, CB1R, located presynaptically [52]. Here we have shown that direct chemical activation of CB1R with WIN-55,212-2 results in LTD in both WT and mutant mice to the same degree. In addition, CB1R protein levels are similar in PirBKO and WT. Thus, we conclude that PirB is specifically needed to couple NMDA receptor activation to endocannabinoid retrograde signaling, implying that the regulation of eCB signaling downstream of glutamate receptors may be divided into PirB-independent (Group I mGluRs) and PirB-dependent (NMDA) pathways.

It is noteworthy that in the absence of PirB in pyramidal cells, there is no change in functional synaptic NMDARs, as evidenced by intact I_AMPA_/I_NMDA_ ratio at baseline, similar decay time constants of NMDA currents, as well as unchanged levels of NR2A and NR2B protein as assessed by standard physiological and biochemical protocols [59, 60]. These observations suggest that PirB acts downstream or subsequent to the activation of NMDA receptors. It is known that when PirB intracellular signaling domains (ITIMs) are phosphorylated, SHP1/2 phosphatases are recruited [14, 61]. In addition, proteomic analysis has identified an interaction between PirB and members of the protein phosphatase 2 (PP2) family including calcineurin (PP2B) (Fig. S8 in [62]) known to be critical for LTD expression [55, 63, 64]. It is possible that in the absence of PirB, there may be alterations in the association and/or synaptic localization of key phosphatases needed for LTD, including calcineurin [63], which could in turn alter intracellular signaling cascades downstream of NMDA receptors that are required for eCB production or release.

### A role for PirB in activity-dependent synaptic pruning

PirB-specific NMDAR signaling during LTD could be tightly linked to structural changes at dendritic spines. Two-photon microscopy in organotypic hippocampal cultures has revealed that low-frequency stimulation (LFS) can enhance dendritic spine retractions [19]. The LFS-induced retraction can be blocked with APV, an NMDAR blocker, suggesting that it is an NMDAR-dependent process [19]. In addition, a study in a mouse model of Alzheimer’s disease has shown that intact PirB is needed to activate cofilin [62]; actived cofilin depolymerizes F-actin and promotes spine shrinkage and retraction [55]. It is thought that calcineurin dephosphorylates cofilin to promote its actin depolymerizing activity [55, 65]. Thus, the requirement for PirB and eCBs in generating and sustaining NMDAR-dependent LTD at CA3-CA1 synapses could be an inextricable part of an NMDAR-specific dendritic spine retraction mechanism [19, 66].

Activity-dependent and NMDAR-dependent synaptic weakening and pruning can be induced in hippocampal slices using low-frequency stimulation (e.g., 1 Hz, known to induce LTD) [18, 19, 55], as well as with chemical NMDA-LTD [56]. Here, in WT we find that within an hour of NMDA-LTD there is a decrease in mEPSC frequency (Fig. 5), as shown previously [57] and consistent with a loss of excitatory synapses. However, in Pyr-KO the NMDA-LTD-dependent decrease in mEPSC frequency is absent, suggesting that when PirB is deleted from pyramidal cells a signaling cascade needed for synaptic elimination is disengaged. In addition, the effect of postnatal PirB deletion on mEPSC frequency is evident even at baseline, prior to NMDA-LTD induction. Together, these observations suggest that without PirB, CA3 to CA1 synapses become resistant to activity-dependent pruning, growing in density to 50% greater than that in adult WT. Note that these observations here in hippocampus are consistent with previously observed elevated mEPSC frequency and spine density in L5 and L2/3 pyramidal neurons of visual cortex arising from impaired activity-dependent synapse pruning during the developmental critical period [15–17].

### Activity-dependent recruitment of PirB and downstream effectors—a model

Here we have shown that PirB is part of an eCB signaling cascade that is recruited by activity, and in its absence, synapses cannot weaken. We propose that PirB function is needed for synapses to adjust and maintain their state to reflect their history of neural activity, while retaining their capacity for further potentiation. The requirement for PirB in bidirectional Hebbian synaptic plasticity also suggests that PirB contributes to the phenomenon of metaplasticity [67]. These considerations imply that changes in neural activity should engage PirB signaling. PirB is an MHC class I (MHCI) receptor, both in neurons and in immune cells [13, 14, 62]. MHCI molecules were discovered in an in vivo expression screen searching for genes regulated by neural activity [68]. Moreover in mice lacking stable MHCI surface expression, hippocampal LTD is absent and LTP is enhanced [54, 69], similar to what is seen here in PirB null mice and consistent with the fact that MHCI molecules bind to PirB. In vitro, MHCIs are also needed for proper activity-dependent scaling of hippocampal synapses [70].

In addition to MHCI proteins, myelin proteins including Nogo-A, OMgp and MAG have been shown to bind to PirB [71]. These ligand-receptor interactions elicit growth cone collapse in vitro [71], and possibly in vivo [72]. In the context of learning and plasticity, multiple independent studies have implicated all three myelin based proteins in negative regulation of ocular dominance plasticity [73], learning processes during fear extinction [74], and synaptic plasticity [75] in adult animals. Even though most of these effects seem to be mediated via Nogo-receptor 1 (NGR1) [73–75], an OMgp-PirB interaction has been implicated in the negative regulation of hippocampal LTP in adult mice [75]. It would be interesting to know if Nogo-A, OMgp, or MAG levels are regulated by neural activity, as is the case for the MHC class I ligands. Together, our current findings suggest a model (Fig. S11) in which PirB signaling is modulated by activity-dependent changes in MHCI levels, which in turn affect intracellular signaling cascades downstream of NMDA receptors required for LTD and eCB production and release.

## Electronic supplementary material

Supplemental Information
