# Supplementary material for: Activity-dependent modulation of hippocampal synaptic plasticity via PirB and endocannabinoids
Source: Mol Psychiatry. 2018 Apr 18;24(8):1206–19. doi: 10.1038/s41380-018-0034-4 (PMC6372352; doi:10.1038/s41380-018-0034-4)
Supplement: Supplementary file 1 — Supplemental Information [file 41380_2018_34_MOESM1_ESM.pdf]

Activity-dependent modulation of hippocampal synaptic plasticity via PirB and endocannabinoids

Maja Djurisic, Barbara K. Brott, Nay L Saw, Mehrdad Shamloo, Carla J. Shatz

Supplementary Information

Supplementary Materials and Methods

Experimental Model and Subject Details

The mouse lines used in this study were generated by crossing PirBfl/fl line with an appropriate Cre-deleter line. PirBfl/fl mice were generated as described previously (1): 129 J1 ES cells derived from agouti 129S4/SvJ mice were electroporated with a construct containing loxP sites flanking exons 10-13 of PirB. These exons code for the transmembrane domain of PirB, as well as part of the intracellular domain encompassing signaling immunoreceptor tyrosine-based inhibitory motifs (ITIMs). Cre-mediated excision of exons 10-13 in PirBfl/fl mice generates a truncated protein unable to signal, as shown previously by anti-phosphotyrosine immunoprecipitation experiments (1). To generate mice with germline deletion of PirB (PirB$^{-/-}$), a deleter strain targeting Cre-recombinase expression to mouse embryos via adenovirus EIIa promoter (B6.FVB-TGN(EIIa-cre)C5379Lmgd, Jackson) was crossed to PirBfl/fl mice. Heterozygote sibling matings were then used to generate both control WT (PirB+/+) and a homozygous PirB$^{-/-}$ line on the same mixed C57Bl/ 6 x SV/ 129J genetic background (1).

Postnatal excision of PirB from forebrain pyramidal cells and CA3 region of hippocampus was accomplished by crossing PirBfl/fl line with CamKII-Cre (B6.Cg-Tg(Camk2a-cre)T29-1Stl/J; JAX 005359; (2)) or Grik4-Cre (C57BL/6-Tg(Grik4-cre)G32-4Stl/J; JAX:006474; (3)) deleter lines. In these Cre lines, ectopic Cre expression in gametes is reported (see JAX datasheets), resulting in peripheral

somatic deletion of floxed alleles in offspring in addition to brain-specific excision. To ensure that only mice with brain-specific deletion of PirB were used in experiments, genotyping was designed to differentiate fully deleted PirB vs. floxed PirB vs. WT PirB alleles in peripheral somatic tissue (ear punch) for each mouse. Only Cre+ mice with floxed PirB alleles or WT PirB alleles, but not fully excised PirB alleles in the peripheral somatic tissue, were used for experiments. This protocol was employed in addition to recommended breeding strategies for each line designed to minimize the effect of ectopic excision in gametes.

A reporter line, Ai14TdTomato, was used to confirm the expected pattern of Cre expression in CamKII-Cre and Grik4-Cre lines. Ai14TdTomato, a gift from Dr. Liqun Luo, is available as B6;129S6-Gt(ROSA)26Sor$^{tm14(CAG\text{-}tdTomato)Hze}$/J, JAX: Stock No:007908, from Jackson Laboratories (Bar Harbor, ME). Ai14TdTomato mice were crossed to CamKIIa-Cre+ or Grik4-Cre+ mice for one generation, and the pattern of TdTomato expression was assessed in Cre+ mice of the F1 generation. Previous studies have shown that excision of PirB from PirBfl/fl by Cre recombinase under the control of the Ubc promoter occurs within one week, accompanied by a complete loss of PirB protein after about 3 weeks from the onset of Cre recombinase expression (e.g. Figure 1, (4)). Experiments reported here with conditional postnatal PirB deletion were all performed in mice older than P90, weeks to months after Cre expression, at which point PirB protein should be fully removed from targeted cell types.

Behavior: Delayed match-to-place (DMP) dry maze

A DMP dry maze test was used to assess spatial working/episodic-like learning and memory. The dry maze is thought to measure similar learning abilities to the original DMP water maze (5, 6). It was conducted using a modified Barnes (dry) maze apparatus (7) consisting of a 122-cm diameter circular platform with 40 escape holes, each with a diameter of 5 cm placed along three rings of varying distances from the center of the platform (Fig. 1a). The outer ring has 16 holes at 50 cm from the center, the middle ring has 16 holes at 35 cm from the center, and the inner ring has eight holes at 20 cm from the center. An escape box was

attached to one of these holes and all holes were left uncovered. Bright overhead lighting (750 lux) and air turbulence created by fans were used to create aversive conditions that would encourage mice to seek out the target hole. Visual cues were placed on all four sides of the maze. Mice were given a series of four trials with 2 minute inter-trial intervals (ITI); the maximum duration of each trial was 90 sec. For each trial, mice were placed in different locations at the edge of the maze and held under a dark cover to prevent directional bias. After 10 sec, the cover was removed and the trial started. The distance from the release point to the escape box was the same within a day. The trial ended if a mouse entered the escape box before the end of the 90 sec. Mice unable to find the escape box were led to it by the experimenter and allowed to enter. As soon as the mouse entered the escape box, the noise was turned off and the mouse remained within the box for 10 sec before being returned to its home cage. Mice were habituated to the escape box for 1 minute on the first day of testing. During the initial two days of testing the escape box was marked by an elevated ping-pong ball (Visual Training protocol) to help mice learn the task, and also enable the experimenter to detect potential gross sensorimotor or visual deficits. Subsequently the ping-pong ball was removed, and mice were tested for five additional days (Hidden Escape Hole protocol). The location of the target escape hole was moved each day during this testing period, while all other parameters remained unchanged. The maze was cleaned with 70% alcohol between each trial. All data was recorded using Ethovision (Noldus Information Technology, Wageningen, The Netherlands). Parameters measured were escape latency, distance moved, and velocity. Mice with low mobility were excluded from the analysis prior to un-blinding.

Hippocampal physiology

Adult hippocampal slice preparation.

Acute hippocampal slices were made from mice older than P90, when hippocampal circuitry is fully mature in terms of ionic conductances (8, 9), synaptic plasticity mechanisms (10, 11), and the eCB retrograde signaling system (12, 13).

Slices were prepared using a "protective recovery" method (14). This approach minimizes excitotoxicity and hypoxia. Mice were overdosed via intraperitoneal injection of Ketamine HCl (KetaVed, VEDCO; 0.4 mg/g body weight), xylazine HCl (AnaSed, AKORN; 0.04 mg/g body weight), and acepromazine maleate (AceproJect, Henry Schein; $6x10^{-4}$ mg/g body weight) cocktail. Under deep anaesthesia, mice were perfused transcardially with ice-cold carbogenated ACSF (~3.5 ml/min), then decapitated, and the brains gently but quickly extracted from the scull and immersed in ice-cold N-methyl-d-glucamine (NMDG)-ACSF (see below) for 1 min. To ensure proper slicing angle for transverse hippocampal slices, a 60 degree wedge centered at the midline was cut out from the rostral end of the forebrain. Hemispheres were separated, and each was glued cut side down onto a mounting disk. The disk carrying the hemispheres was immersed in a chamber containing ice-cold carbogenated NMDG-ACSF; a block of 5% agar was glued behind the hemispheres to provide additional support during cutting. Transverse sections (400 µm) were cut with a vibrating microtome (Leica VT 1000S). Hippocampal sections with cortex attached were transferred to a recovery chamber containing carbogenated ACSF at room temperature, and incubated for 2-4 hours before recording. This protocol ensures that slices from adult mice remain healthy and viable for long periods. ACSF (in mM): 125 NaCl, 26 $NaHCO_3$, 2.3 KCl, 1.26 KH_2PO_4, 1.3 $MgCl_2$, 2.5 $CaCl_2$, 25 glucose. NMDG-ACSF (in mM): 135 NMDG, 1 KCl, 1.2 KH_2PO_4, 1.5 $MgCl_2$, 0.5 $CaCl_2$, 20 choline bicarbonate, 10 glucose.

Hippocampal synaptic plasticity

LTP and LTD experiments at CA3 - CA1 synapses were performed using extracellular field recordings. A concentric bipolar stimulating electrode (FHC #CBABD70, Bowdoin, ME) was positioned at the Schaffer collateral bundle at the border of CA2 and CA1; a low-resistance recording microelectrode – an Ag/$AgCl_2$ wire inserted into a patch-pipette filled with ACSF- was placed in the middle of CA1 *st. radiatum* about 200-300 µm away from the stimulating electrode. Test pulses were applied at 0.033 Hz. The LTP induction paradigm consisted of four 100 Hz tetani delivered at 15 s intervals; each tetanus consisted of 100 pulses. For LTP, stimulation intensity used to generate field excitatory postsynaptic potentials (fEPSPs) during

both baseline and induction stimulation was set at 30% of the constant current needed to elicit a population spike. LTD was induced with a single low-frequency stimulation (LFS) epoch consisting of 900 pulses delivered at 1Hz; in this case stimulation intensity was set at 50% of spiking threshold both during baseline and induction. These stimulation and induction paradigms are used extensively (15, 16). For LTP and LTD measurements in Pyr-KO/Pyr-WT and CA3-Pyr-KO/CA3-Pyr-WT mice, baseline stimulation consisted of a pair of pulses separated by 80 ms. Paired-pulses were delivered at 0.033 Hz; slopes of resulting fEPSPs were used to calculate paired-pulse ratio (PPR) before and after plasticity induction: PPR= fEPSP2 slope/fEPSP1 slope. For chemical LTD, bath application of WIN-55,212-2 (5 µm in ACSF) for 15 min, DHPG (100 µm in ACSF) for 15 min, and NMDA (20 µm in ACSF) for 3 minutes was used. After either LTP or LTD induction, fEPSPs were recorded for 60-90 minutes. Throughout the experiment, a linear fit was used to quantify fEPSP slopes between 20-60% of peak amplitude, ensuring minimal contamination from the fiber volley.

To isolate excitatory synaptic inputs, all LTP and LTD measurements were done in the presence of 100 µm picrotoxin – a GABAA receptor blocker - in the bath. Picrotoxin eliminates both synaptic and tonic GABA transmission (17), as well as heterosynaptic effects driven by endocannabinoid-dependent inhibitory-LTD (I-LTD) on the threshold for LTP at neighboring CA3-CA1 synapses (18). These experimental conditions allowed us to assess the effect of PirB deletion on homosynaptic plasticity specifically at excitatory CA3-CA1 synapses. To prevent epileptic activity, the CA3 region was cut off prior to each experiment. Picrotoxin also increases excitability of hippocampal slices via blockade of tonic inhibition, resulting in lower spiking threshold; consequently, the stimulation needed for 30% or 50% of the spiking threshold was readjusted after addition of picrotoxin. In a small number of recordings, slice health deteriorated and slices became hyper-excitable, as evident by the appearance of a population spike; these experiments were discarded.

Flow rate of ACSF through the recording chamber was 2 ml/min. Signals were acquired with a DP304 Differential amplifier (Warner Instruments, Hamden, CT), DigiData 1322A digitizer, using Clampex software (Molecular Devices LLC, Sunnyvale, CA), and analyzed with Clampfit module.

Intracellular CA1 recordings.

The whole-cell patch-clamp technique was used to record evoked synaptic currents, mEPSCs, and action potentials from CA1 cells. Healthy CA1 cells were visualized using IR-DIC illumination in an Olympus BX51WI microscope via a 60X water immersion objective coupled with an additional 2X zoom lens (120X final magnification). Only cells from the top of the pyramidal layer with pyramidal-shaped somata that had a visible apical dendrite extending into the *st. radiatum* were selected for recordings. All measurements were performed in standard ACSF solution, at 30-32°C. For AMPA/NMDA ratio, PPF measurements, and mEPSCs, patch-clamp electrodes (2-6 MΩ) were filled with Cs-based intracellular solution (in mM): 108 Cs-methane sulfonate, 9 HEPES, 4 $MgCl_2$, 1 EGTA, 5 Mg_2ATP, 1 NaGTP, 4 QX-314, 15 TRIS phosphocreatine, pH 7.3 with CsOH. For intrinsic excitability measurements in current-clamp mode, the intracellular solution was (in mM): 115 CH_3KO_3S, 20 KCl, 10 HEPES, 0.2 EGTA, 4 Mg_2ATP, 0.3 NaGTP, 10 Na_2 phosphocreatine, pH 7.3 with KOH. After gaining access into the cell, the pipette solution equilibrated with the cell contents for 5-10 minutes before the recording.

AMPA currents were recorded at -70 mV holding membrane potential in the presence of 4 mM Mg^{2+} and 10 µm SR 99535. Subsequent addition of 10 µM CNQX in the bath enabled NMDA current recordings at +40 mV. AMPAR and NMDAR decay currents were fitted with weighted single or double exponential functions $f(t) = \frac{1}{\tau 0} * (\sum_{i=1}^{n} fi * exp\left(-\frac{t}{\tau i}\right) + C$ in Clampex software to assess tentative changes in kinetics between different genotypes. f_i is a relative contribution of a τ_i component in total I_{AMPA} or I_{NMDA}. For PPF, I_{AMPA} was measured in the presence of 50 µm AP-5, 10 µm SR 99535 and 4 mM Mg^{2+}. Intrinsic excitability was assessed in the complete blockade of all synaptic transmission (10 µM CNQX, 50 µm AP-

5, 10 µm SR 99535). mEPSCs were isolated pharmacologically using 1 µm TTX, 50 µm AP-5 and 10 µm SR 99535; in addition, $MgCl_2$ in the bath was increased to 4 mM.

DSI of evoked IPSCs in CA1 neurons was induced by 5 s depolarization from -70 mV to -0 mV. IPSC amplitude was interrogated by stimulation in *st. radiatum* at 0.33 Hz. Change in peak amplitude of IPSCs due to depolarizing pulse was determined relative to IPSC amplitude preceding the pulse. Recordings were made in standard ACSF in the presence of 10 µm CNQX; application of 10 µm SR95531 at the end of the experiment confirmed that the measured currents were purely of GABA receptor origin. Intracellular solution was (in mM): 135 mM CsCl, 10 mM Hepes, 4 mM QX-314, 1 mM EGTA, 4 mM Mg-ATP, 0.4 mM Na-GTP, pH 7.4 with NaOH.

Series resistance was 9-20 MΩ. Signals were acquired with an AxoPatch 200B, DigiData 1322A digitizer or DigiData 1550 A with HumSilencer, using Clampex software (Molecular Devices LLC, Sunnyvale, CA). Evoked currents and spikes were analyzed with Clampfit module. Analysis of mEPSCs was done using MiniAnalysis software, ver. 6.0.7 (Synaptosoft). Prior to analysis, mEPSCs were filtered with a 2 kHz, low-pass Elliptical filter. Synaptosoft parameters were set to: threshold - 8 pA, search for a local maximum - 20,000 µs; time before a peak for baseline - 5,000 µs; period to search a decay time - 5,000; fraction of peak to find a decay time - 0.5; period to average a baseline - 2,000 µs; area threshold - 10; number of points to average for peak - 3; direction of peak, negative.

List of Drugs and Vendors

SR 99535 (SR 95531 hydrobromide) –Tocris Bioscience, #1262; CNQX (CNQX disodium salt) - Tocris Bioscience, #1045; D-AP5 - Tocris Bioscience, #0106; Picrotoxin - Sigma Aldrich. #P1675; (RS)-3,5-DHPG - Tocris Bioscience, #0342; WIN 55,212-2 mesylate - Tocris Bioscience, #1038; NMDA, Tocris Bioscience, #0114; AM251 - Cayman Chemicals, #71670, TTX (Tetrodotoxin citrate) – Tocris Bioscience, #1069, Ro 25-6981 - Tocris Bioscience,# 1594.

Histology

Tissue preparation.

Adult mice (>P90) received an overdose of sodium pentobarbital (>86 mg/kg) and sodium phenytoin cocktail (Beuthanasia-D, Merck; >11 mg/kg) via intraperitoneal injection. Animals were transcardially perfused with ice-cold phosphate-buffered saline (PBS) followed by ice-cold 4% (wt/vol) paraformaldehyde (PFA) in PBS. Brains were removed from the skull and further post-fixed overnight in 4% (wt/vol) PFA at 4ºC.

For mice from the reporter line Ai14 TdTomato crossed to either CamKIIa-Cre or Grik4-Cre deleter lines, separate hemispheres were sectioned at 40 µm, one in the coronal plane, the other in the sagittal plane, using a Leica VT 1200S system (Leica Microsystems Inc, Bannockburn, IL). Sections were mounted on subbed slides and cover-slipped using ProLong® Gold antifade reagent (Invitrogen Corp., Carlsbad, CA) and No. 1.5 coverslips (VWR International, LLC, West Chester, PA) to visualize the pattern of TdTomato fluorescence.

Immunostaining.

For CB1R and VGlut1 immunostaining, fixed brain tissue (per above) was sectioned at 10 µm through the hippocampal region. Immunohistochemistry was performed on free floating sections. After cutting, sections were briefly rinsed in PBS, permeabilized for 2 x 10 min in 0.2% TX-100 in PBS, and incubated for 1 hr in blocking solution (0.3% TX-100, 5 % goat serum in PBS) at room temperature. Sections were then incubated with primary antibodies overnight at 4ºC on a horizontal shaker: rabbit anti-cannabinoid receptor 1 C-terminus (anti-CB1R-CT, diluted to 1:200, gift from Dr. Ken Mackie, Indiana University, Bloomington, IN) and a guinea-pig anti-vesicular glutamate transporter 1 (anti-VGlut1, 1:5000, #AB5905, Chemicon®, EMD Millipore, Billerica, MA). Sections were then rinsed in 1% goat serum in PBS (3 x 15 min), and incubated for 45-60 min at room temperature in blocking buffer containing secondary antibodies:

goat anti-rabbit IgG conjugated with Alexa Fluor 488 (1:1000, Invitrogen-Molecular Probes, Eugene, OR) to detect anti-CB1R-CT, and goat anti-guinea pig IgG conjugated with Alexa Fluor 647 (1:1000, Invitrogen-Molecular Probes, Eugene, OR) to detect anti-VGlut1. Sections were then thoroughly rinsed in PBS (3 x 15 min to 3 x 30 min), mounted on gelatin-coated slides and coverslipped using ProLong® Gold antifade reagent (Invitrogen Corp., Carlsbad, CA) and No. 1.5 coverslips (VWR International, LLC, West Chester, PA).

Confocal microscopy and image processing.

Fluorescent sections containing hippocampal regions of interest were imaged with an SP2 or SP8 confocal microscope (Leica Microsystems, Buffalo Grove, IL), using 10x/0.4 NA dry objective or 63x/1.40 NA oil-immersion. Composite micrographs were assembled using Photomerge tool in Adobe Photoshop CS4 (Adobe Systems, San Jose, CA). Immunofluorescent images in Fig.4 and Fig. S5. were generated as follows: original Leica microscopy files were imported into Fiji (19) as separate red (VGlut1) and green (CB1R) channel via Bio-Formats Importer plugin (20); each channel was processed to subtract background (Process => Substract Background), and remove laser speckle (Process => Noise => Despeckle); these preprocessed VGlut1 and CB1R channels were then merged (Image => Color => Merge Channels) to visualize co-localization between the two signals as yellow pixels.

Western Blots

For CB1R and NR1 Western blots, cortices were isolated from P90 or older Pyr-WT and Pyr-KO mice (five mice per genotype), and whole-cell lysates were prepared by douncing ten times in a dounce homogenizer in 2 ml Lysis Buffer (150 mM NaCl, 50 mM Tris, 0.25 % sodium deoxycholate, 1% NP-40, 1 mM EGTA, 1 mM PMSF, 1X Pefabloc (Roche)), followed by centrifuging at 12,000xg at 4ºC for 10 minutes. Protein concentrations of supernatants were evaluated by Bradford protein assay (Bio-Rad). Samples were then heated to 85ºC in 1X NuPAGE LDS Sample Buffer (Invitrogen) + 1% b-2 mercaptoethanol, for 3 minutes,

and then electrophoresed on a 10% SDS-PAGE gel. Gel proteins were transferred to Immobilon-P PVDF transfer membrane (Millipore), and Western blotted with antibodies to CB1R (Indiana University at Bloomington, IN; see also (21)), NR1 (Cell Signaling, 5704S), NR2A (Millipore, 07-632), NR2B (Cell Signaling, 14544S) or GAPDH (Abcam, ab9485). Western blots were developed by incubation with anti-Rabbit-HRP secondary antibodies, and then with ECL reagents (GE Healthcare). JPEG files of film exposures were made, and gel bands were quantified using ImageJ software. Values for CB1R, NR1, NR2A and NR2B were normalized to GAPDH levels for each sample.

Supplementary Figure Legends

Supplementary Figure S1. Average savings during DMTP is larger in PirB-/- vs. WT mice.

A) Bar-graphs of average savings in distance between trial 1 and trial 4 for the three non-cued days. Superimposed symbols are savings from individual mice. Note larger distance savings in PirB-/- (~ 200 cm) than in WT mice (~ 30 cm), some of which never acquired the task, $p < 0.01$; U-test. **B)** Bar-graph of average savings in elapsed time between T4 and T1 confirms significantly better performance of PirB-/- mice. * $p < 0.05$; U-test.

Supplementary Figure S2. Synaptic properties and excitability of CA1 pyramidal cells at baseline are similar between WT and PirB-/-, but input-output curves suggest an increase in excitatory synaptic transmission in PirB-/-.

A) Example voltage-clamp traces of evoked I_{AMPA} and I_{NMDA} (left panel). I_{AMPA} was measured at -70 mV in the presence of high magnesium (4 mM) to isolate AMPAR current only. Application of CNQX (10 µM) completely abolished the current. I_{NMDA} was recorded at +40 mV, after blockade of I_{AMPA} with 10 µM CNQX. All recordings were made in the presence of GABAR blocker SR95531 (10 µM). Plot of AMPA/NMDA ratio (right panel) reveals no difference between WT (n = 15 cells/ 5 mice) and PirB-/- (n = 14 cells/ 5 mice; p = 0.65, U-test). **B)** Example voltage-clamp recording of evoked I_{AMPA} from WT (red)

and PirB-/- (black) CA1 pyramidal cells in the presence of 50 µm APV and 10 µm SR95531 (left panel). Holding potential was -70 mV. The traces are scaled to peak for easier comparison. Blue line is a monoexponential fit of the I_{AMPA} decay current. Right panel: a bar-graph of I_{AMPA} time constant from WT (n = 14 cells/5 mice) vs. PirB-/- (n = 15 cells/4 mice) CA1 neurons shows no statistically significant difference; p = 0.621, U-test. **C**) Top panel: example voltage-clamp recording of evoked I_{NMDA} from WT (red) and PirB-/- (black) CA1 pyramidal cells in the presence of 10 µm CNQX and 10 µm SR95531. Holding potential was +40 mV. The traces are scaled to peak for easier comparison. Blue line is a double exponential fit of the I_{NMDA} decay current. Bottom panels: Decay time constants of I_{NMDA} and contribution of the fast and slow decay time constants to I_{NMDA} (right panel), suggest that the fraction of NR2B subunits in NMDA receptors is not increased in PirB-/-. WT (n = 16 cells/6 mice) vs. PirB-/- (n=17 cells/6 mice): τ_{slow}: p = 0.4; τ_{fast}: p = 0.62; % slow component: p = 0.86; %fast component: p = 0.86; U-test. **D)** Example voltage-clamp recording of evoked I_{AMPA} from WT (red) and PirB-/- (black) CA1 pyramidal cells at 0, 20, 50, 150 and 300 ms after the first stimulus, in the presence of 50 µm APV and 10 µm SR95531 (left panel). Holding potential was -70 mV. Paired-pulse facilitation (PPF) was calculated as amplitude of each I_{AMPA} current relative to the amplitude of I_{AMPA} at 0 ms. The amount of PPF does not differ significantly between WT (n=14 cells/ 3 mice) and PirB-/- (n= 12 cells/3 mice), p = 0.092, 2-way RM Anova (right panel). **E)** Example membrane voltage traces from CA1 pyramidal neurons recorded in current-clamp mode under conditions of complete blockade of synaptic transmission (left panel). Current injections ranged from 50 to 600 pA. Under these conditions, threshold for spiking is dependent only on intrinsic membrane properties. For clarity, only a subset of voltage traces from the series is shown. Holding potential: -70 mV. Plot of instantaneous firing rate (Hz) measured at holding potential of V_h = -70 mV vs. injected current (pA) in current-clamp conditions (right panel). No statistically significant difference was observed for instantaneous firing rate between WT (n = 18 cells/1 mouse) and PirB-/- (n = 17 cells/6 mice), p=0.933, 2 Way RM Anova. **F)** Top panel: Average synaptic input-output plot, a measure of the strength of excitatory synaptic transmission, assessed as fEPSP slope (mV/ms) vs. stimulus intensity (µA). PirB-/- (n=16 slices/10 mice) have on average significantly steeper I/O curves, suggesting stronger synaptic transmission than WT

(n=17 slices/13 mice); p<0.001, 2 way RM Anova. Data is plotted along x-axis in bins from 10-30 µA, 30-50 µA, and 50-90 µA. Bottom panel: as above, but showing I/O curves calculated as fEPSP slope (mV/ms) vs. fiber volley (FV) amplitude (mV). Data is plotted in bins of FV amplitude from 0.01-0.05 mV, 0.05-0.1 mV, and 0.1-0.15 mV. PirB-/- (n=16 slices/10 mice) vs. WT (n=17 slices/13 mice); p=0.009, 2 way RM Anova.

Supplementary Figure S3. Confirmation of Cre expression in the CamKIIa-Cre line

CamKIIα-Cre mice were crossed to the Ai14 Td Tomato reporter line (TdTomato fluorescence is a readout for Cre expression). **A)** Sagittal section through hippocampus at P100 shows TdTomato fluorescence (red) in CA1 and CA3 pyramidal neurons, and dentate gyrus (DG). Calibration bar: 0.5 mm. **B)** Higher magnification of CA1 shows majority of neurons expressing Cre recombinase. **C)** CA3 region and **D)** Dentate Gyrus (DG). **(B, C, D)** Calibration bar: 20 µm.

Supplementary Figure S4. NMDAR specific blocker D-AP5 abolishes LTP and LTD in both Pyr-WT and Pyr-KO hippocampal slices.

A) LTP is abolished with D-AP5 in both Pyr-WT (100.94 ± 0.57 %, n=15 slices/6 mice) and Pyr-KO (101.72 ± 0.78 %, n=11 slices/5 mice); p=0.297, 2-Way RM Anova. **B)** LTD also abolished in D-AP5. Pyr-WT LTD: 108.4 ± 0.9 % (n=9 slices/3 mice). Pyr-KO: 108.6 ± 0.65 % (n=10 slices/3 mice); p=0.306, 2-Way RM Anova.

Supplementary Figure S5. Paired-pulse ratio differs between Pyr-WT and Pyr-KO during both LTD and LTP.

A) Cumulative average of paired-pulse ratios (PPR) during LTD. Pyr-WT LTD: 1.10 ± 0.01 (n=12 slices, 6 mice) vs. Pyr-KO LTD: 0.970 ± 0.003 (n=16 slices, 6 mice); p<0.001. **B)** Cumulative average of PPR during LTP. Pyr-WT LTP: 0.999 ± 0.16 (n=8 slices, 5 mice) vs. Pyr-KO LTP: 0.927 ± 0.004 (n=8 slices, 3

mice), p<0.001. Test: 2-Way RM Anova. Values from 20-90 min post-induction were compared. **Insets:** fEPSP example traces measured during LTD or LTP to derive PPR. Traces in gray: average of 30 individual traces at baseline; traces in black: average of 60 individual traces after LTD (A) or LTP (B) induction. LTD expression (A) in Pyr-WT, is accompanied by an increase in PPR as expected; in Pyr-KO PPR *decreases* following LTD induction, resulting in LTP instead of LTD (see Fig. 2B, 2E). C) Bar-graph comparing PPR between Pyr-WT and Pyr-KO at baseline, prior to either LTD or LTP induction. PPR is calculated by dividing absolute values of slopes of fEPSP2/fEPSP1. No difference in PPR between the two genotypes is observed prior to LTD or LTP. PPR before LTD: Pyr-WT: 1.23 ± 0.05 (n=12 slices, 6 mice) vs. Pyr-KO: 1.26 ± 0.04 (n=16 slices, 6 mice); p=0.374. PPR before LTP: Pyr-WT: 1.68 ± 0.08 (n=8 slices, 5 mice) vs. Pyr-KO: 1.61 ± 0.14 (n=8 slices, 3 mice), p=0.063. Test: 2-Way RM Anova; n.s. = not significant. PPR values prior to LTD are smaller than before LTP because stimulation intensity used for LTD was 50% of firing threshold instead of 30% used for LTP (see Supplementary Materials and Methods).

Supplementary Figure S6. CB1R and VGlut1 in CA1 region from Pyr-WT and Pyr-KO

A) Pyr-WT immunofluorescence shown for CB1R (green; left panel), VGlut1 (red; right panel) and both signals together (middle panel; yellow pixels indicate presence of both CB1R+VGlut1). CB1R immunofluorescence is present in axonal fibers and boutons in *stratum pyramidale* (SP) near CA1 somata, as well as in *stratum radiatum* (SR), and *stratum oriens* (SO). VGlut1 signal is abundant in *st. radiatum*, and mostly absent from *st. pyramidale*, as expected. **B**) As in A, but in Pyr-KO. **C**) No primary-controls shown using same imaging conditions as in A and B. Scale bar: 10 µm.

Supplementary Figure S7. DSI of evoked IPSCs is intact in Pyr-KO

A) Traces of IPSCs in Pyr-WT and Pyr-KO before and after 5 s depolarization to 0 mV. IPSCs were measured at -70 mV in the presence of 10 µm CNQX. Each trace is an average of 10 traces taken before and immediately after the pulse. **B)** A plot of a cumulative average of IPSC amplitudes before and after the

5 s pulse to 0 mV. DSI is defined as a 10 sec decrease in the peak amplitude in IPSCs, relative to baseline, seen immediately after the 0 mV pulse (22). There was no difference in DSI between Pyr-WT (66.7 ± 3.4 %, n = 12 cells/3 mice) vs. Pyr-KO (64.2 ± 6.8 %, n = 16 cells/3 mice), p=0.694, 2-Way RM Anova, for the first three data points after the 0 mV pulse. **C)** In a subset of cells from one mouse each from Pyr-WT and Pyr-KO, CB1R dependence of DSI was confirmed by recording in the presence of 5 µM AM251, a CB1R blocker. Traces of IPSCs as in A, but in the presence of AM251: a difference in IPSC amplitude seen before vs. immediately after the depolarizing pulse is abolished. **D)** A plot of a cumulative average of IPSC amplitudes before and after the 5 s pulse to 0 mV in the presence of AM251. DSI was abolished, as expected, in Pyr-WT (103.70 ± 1.17 %, n = 6 cells), but also in Pyr-KO (103.04 ± 5.72, n = 5 cells), p=0.275, 2-Way RM Anova, for the first three data points after the 0 mV pulse.

Supplementary Figure S8. DHPG-LTD occludes synaptically-induced LTP

A) 1Hz LTD-induction protocol (arrow) following mGluR-dependent chemical with 100 µM DHPG: Pyr-WT: 66.3 ± 0.71 % (n=11 slices/ 4 mice) vs. Pyr-KO: 59.5 ± 1.18 % (n=11slices/4 mice). **B**) PPR after addition of DHPG (left) and then following 1 Hz synaptically induced LTD: Pyr-WT: 2.55 ± 0.45 (n=11 slices, 4 mice) vs. Pyr-KO: 2.90 ± 0.40 (n=11 slices, 4 mice); p=0.517. Test: 2-Way RM Anova.

Supplementary Figure S9. NMDAR function and subunit composition is comparable between Pyr-WT and Pyr-KO

A) Western Blot analysis of NR1 levels relative to GAPDH, for Pyr-WT and Pyr-KO. Upper - image of a representative gel showing NR1 specific and GAPDH specific bands. Lower – bar graph of relative NR1 protein abundance in Pyr-WT (0.83 ± 0.03, n=5) vs. Pyr-KO (0.78 ± 0.02, n=5), p=0.15, U-test. **B)** Western Blot analysis of NR2A and NR2B levels relative to GAPDH, for Pyr-WT and Pyr-KO. Upper - image of a representative gel showing NR2A and NR2B specific and GAPDH specific bands. Lower left – bar graph of relative NR2A protein abundance in Pyr-WT (0.81 ± 0.07, n=4) vs. Pyr-KO (0.82 ± 0.04, n=4), p=0.89, U-test. Lower right - bar graph of relative NR2B protein abundance in Pyr-WT (1.03 ± 0.06, n=4) vs. Pyr-

KO (1.01 ± 0.02, n=4), p=0.69, U-test. **C)** I_{AMPA}/I_{NMDA} ratio is comparable between Pyr-WT and Pyr-KO. Example voltage-clamp traces of evoked I_{AMPA} and I_{NMDA} (upper panel) for Pyr-WT and Pyr-KO. I_{AMPA} was measured at -70 mV in the presence of high magnesium (4 mM) to isolate AMPAR current. Application of 10 µM CNQX, an AMPAR antagonist, completely abolished the current measured at -70 mV. I_{NMDA} was next recorded at +40 mV. GABAAR blocker SR95531 (10 µM) was present in the bath throughout the recording. Peak amplitudes of I_{AMPA} and I_{NMDA} were used to calculate AMPA/NMDA ratio (bottom panel); the bar graph reveals no difference between Pyr-WT (n = 13 cells/ 6 mice) and Pyr-KO (n = 5 cells/ 3 mice; p = 0.775, U-test). **D)** I_{NMDA} decay (data from C) was fitted with weighted double exponential function, and fast (fast τ1 – top left panel) and slow decay time constants (slow τ2 – top right panel) of I_{NMDA}, and their respective contributions to the total I_{NMDA} (bottom left and right panels) were determined. fast τ1: Pyr-WT (n = 13 cells/6 mice) vs. PirB-/- (n=5 cells/3 mice): p = 0.21; slow τ2, p = 0.059; % fast component: p = 0.173; % slow component: p = 0.25; U-test. **E)** Left - Example voltage-clamp traces of evoked I_{NMDA} before (control) and after application of 1 µm Ro 25-6981 (Ro 25-6981) at +40 mV, in the presence on 1.3 mM Mg. All recordings were made in the presence of GABAR blocker SR95531 (10 µM). Right – Plot of the residual I_{NMDA} after 10 min blockade with 1 µm Ro 25-6981, relative to control. No significant difference is observed between Pyr-WT (51.7 ± 7.2 %, n= 5 cells, 3 mice) and Pyr-KO (63.7 ± 3.3 %, n = 8 cells/3 mice), p = 0.17, U-test.

Supplementary Figure S10. Mice with presynaptic CA3-specific deletion of PirB display normal NMDA-LTD

A) CA3-specific Cre deleter mice (Grik4-Cre; PirB flox/+) were crossed to the Ai14 TdTomato reporter line. TdTomato (red fluorescence) is expressed only when Cre is present. Sagittal sections of adult hippocampus (P100) show Cre recombinase present in CA3 **(B)** and dentate gyrus (DG) excitatory neurons **(C)**, but not in CA1 **(A)**. Calibration bar: 500 µm. SO = stratum oriens, SP = stratum pyramidale, SR = stratum radiatum, SLM = stratum lacunosum moleculare. **D)** NMDA-LTD in CA3-Pyr-WT: 48.5 ± 1.3 % (n=19 slices/7 mice; p<0.001 relative to baseline, one-way Anova) vs. CA3-Pyr-KO: 57.5 ± 1.2% (n=21

slices, 8 mice; p<0.001 relative to baseline, one-way Anova). **Inset:** fEPSP trace examples from CA3-Pyr-WT and CA3-Pyr-KO slices; gray line and black line fEPSP traces are before and after LTD induction, respectively; each trace is average of 30 individual traces taken at baseline or at 75-90 min post-induction. **E)** Average PPR in CA3-Pyr-WT: 1.71 ± 0.13 (n=19 slices/7 mice) vs. CA3-Pyr-KO: 1.66 ± 0.07 (n=21 slices, 8 mice); p=0.7, 2-Way RM Anova. Data averaged from 30-90 minutes.

Supplementary Figure S11. Hypothetical model for PirB and endocannabinoid- NMDA- dependent LTD

A) Bidirectional synaptic plasticity at adult WT CA3-CA1 synapses. **B)** In WT, 1 Hz LTD-induction protocol (1) boosts NMDA-dependent (2) recruitment of eCBs (3) that retrogradely bind to presynaptic CB1 receptors (4), leading to decreased glutamate release (5) and expression of LTD (6). Red arrows illustrate proposed pathway recruited by PirB. **C)** In absence of PirB (PirB null), synapses cannot undergo LTD and instead experience LTP across all induction frequencies. **D)** Following the 1 Hz LTD-induction protocol (1) in PirB null, the NMDA-dependent component (2) of eCB-CBR1 retrograde signaling is not engaged (3, 4) and glutamate release (5) is not affected. PirB is depicted with six Ig-like extracellular domains (blue), and four intracellular ITIM domains (green).

Supplementary References

1. Syken J, Grandpre T, Kanold PO, Shatz CJ. PirB restricts ocular-dominance plasticity in visual cortex. *Science* 2006 Sep 22; **313**(5794)**:** 1795-1800.

2. Tsien JZ, Chen DF, Gerber D, Tom C, Mercer EH, Anderson DJ *et al.* Subregion- and cell type-restricted gene knockout in mouse brain. *Cell* 1996 Dec 27; **87**(7)**:** 1317-1326.

3. Nakazawa K, Quirk MC, Chitwood RA, Watanabe M, Yeckel MF, Sun LD *et al.* Requirement for hippocampal CA3 NMDA receptors in associative memory recall. *Science* 2002 Jul 12; **297**(5579)**:** 211-218.

4. Bochner DN, Sapp RW, Adelson JD, Zhang S, Lee H, Djurisic M *et al.* Blocking PirB up-regulates spines and functional synapses to unlock visual cortical plasticity and facilitate recovery from amblyopia. *Sci Transl Med* 2014 Oct 15; **6**(258)**:** 258ra140.

5. Steele RJ, Morris RG. Delay-dependent impairment of a matching-to-place task with chronic and intrahippocampal infusion of the NMDA-antagonist D-AP5. *Hippocampus* 1999; **9**(2)**:** 118-136.

6. da Silva BM, Bast T, Morris RG. Spatial memory: behavioral determinants of persistence in the watermaze delayed matching-to-place task. *Learn Mem* 2014 Jan; **21**(1)**:** 28-36.

7. Barnes CA. Memory deficits associated with senescence: a neurophysiological and behavioral study in the rat. *J Comp Physiol Psychol* 1979 Feb; **93**(1)**:** 74-104.

8. Maletic-Savatic M, Lenn NJ, Trimmer JS. Differential spatiotemporal expression of K+ channel polypeptides in rat hippocampal neurons developing in situ and in vitro. *J Neurosci* 1995 May; **15**(5 Pt 2)**:** 3840-3851.

9. Vasilyev DV, Barish ME. Postnatal development of the hyperpolarization-activated excitatory current Ih in mouse hippocampal pyramidal neurons. *J Neurosci* 2002 Oct 15; **22**(20)**:** 8992-9004.

10. Yasuda H, Barth AL, Stellwagen D, Malenka RC. A developmental switch in the signaling cascades for LTP induction. *Nat Neurosci* 2003 Jan; **6**(1)**:** 15-16.

11. McHail DG, Dumas TC. Multiple forms of metaplasticity at a single hippocampal synapse during late postnatal development. *Dev Cogn Neurosci* 2015 Apr; **12:** 145-154.

12. Piyanova A, Lomazzo E, Bindila L, Lerner R, Albayram O, Ruhl T *et al.* Age-related changes in the endocannabinoid system in the mouse hippocampus. *Mech Ageing Dev* 2015 Sep; **150:** 55-64.

13. Yasuda H, Huang Y, Tsumoto T. Regulation of excitability and plasticity by endocannabinoids and PKA in developing hippocampus. *Proc Natl Acad Sci U S A* 2008 Feb 26; **105**(8)**:** 3106-3111.

14. Ting J, Daigle, T., Chen, Q., and Feng, G. Acute Brain Slice Methods for Adult and Aging Animals: Application of Targeted Patch Clamp Analysis and Optogenetics. In: Martina M, Taverna, S. (ed). *Patch-Clamp Methods and Protocols*, vol. 1183. Springer: New York, 2014, pp 221-242.

15. Huh GS, Boulanger LM, Du H, Riquelme PA, Brotz TM, Shatz CJ. Functional requirement for class I MHC in CNS development and plasticity. *Science* 2000 Dec 15; **290**(5499)**:** 2155-2159.

16. Dudek SM, Bear MF. Homosynaptic long-term depression in area CA1 of hippocampus and effects of N-methyl-D-aspartate receptor blockade. *Proc Natl Acad Sci U S A* 1992 May 15; **89**(10)**:** 4363-4367.

17. Prenosil GA, Schneider Gasser EM, Rudolph U, Keist R, Fritschy JM, Vogt KE. Specific subtypes of GABAA receptors mediate phasic and tonic forms of inhibition in hippocampal pyramidal neurons. *J Neurophysiol* 2006 Aug; **96**(2)**:** 846-857.

18. Chevaleyre V, Castillo PE. Endocannabinoid-mediated metaplasticity in the hippocampus. *Neuron* 2004 Sep 16; **43**(6)**:** 871-881.

19. Schindelin J, Arganda-Carreras I, Frise E, Kaynig V, Longair M, Pietzsch T *et al.* Fiji: an open-source platform for biological-image analysis. *Nat Methods* 2012 Jun 28; **9**(7)**:** 676-682.

20. Linkert M, Rueden CT, Allan C, Burel JM, Moore W, Patterson A *et al.* Metadata matters: access to image data in the real world. *J Cell Biol* 2010 May 31; **189**(5)**:** 777-782.

21. Wager-Miller J, Mackie, K.. Western Blotting of the Endocannabinoid System. In: Maccarrone M (ed). *Endocannabinoid Signaling Methods and Protocols*, vol. 1412. Springer: New York, 2016, pp 247-254.

22. Wilson RI, Kunos G, Nicoll RA. Presynaptic specificity of endocannabinoid signaling in the hippocampus. *Neuron* 2001 Aug 16; **31**(3)**:** 453-462.

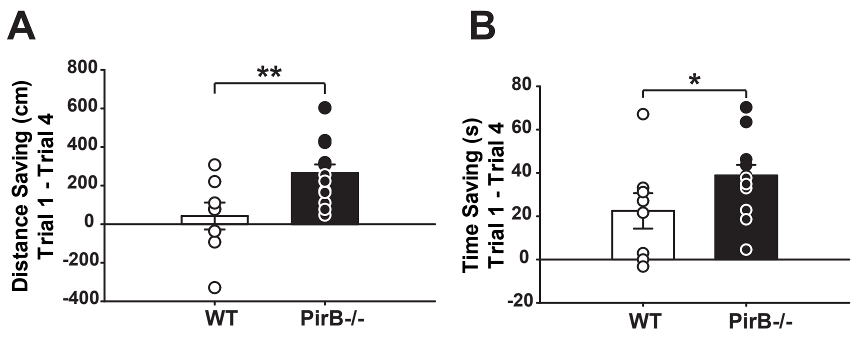
A
Distance Saving (cm)
Trial 1 - Trial 4
800
600
400
200
0
-200
-400
**
WT
PirB-/-
B
Time Saving (s)
Trial 1 - Trial 4
80
60
40
20
0
-20
*
WT
PirB-/-

Figure S1

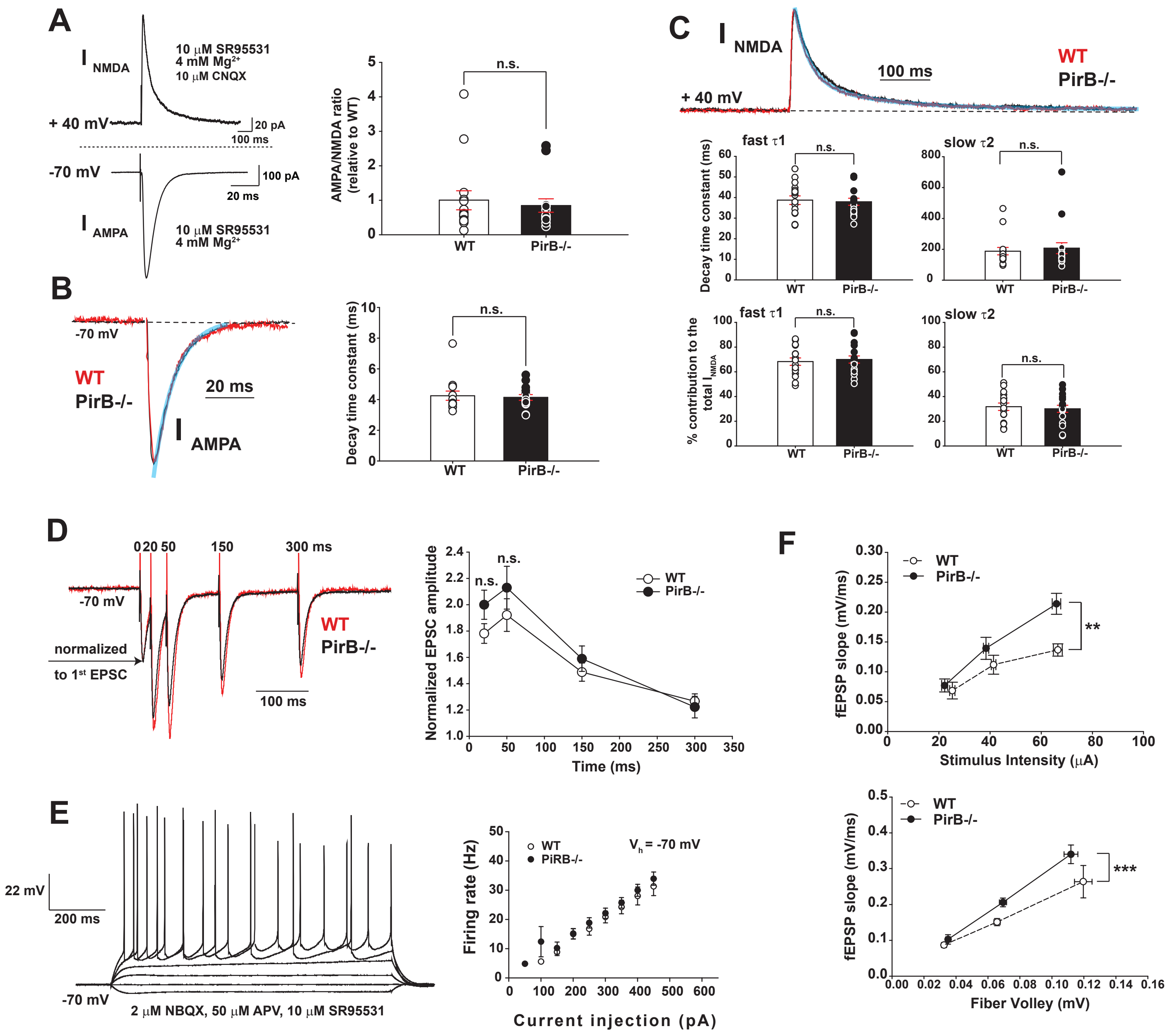
A
I NMDA
10 µM SR95531
4 mM Mg2+
10 µM CNQX
+ 40 mV
20 pA
100 ms
-70 mV
100 pA
20 ms
I AMPA
10 µM SR95531
4 mM Mg2+
AMPA/NMDA ratio (relative to WT)
n.s.
WT
PirB-/-
B
-70 mV
WT
PirB-/-
20 ms
I AMPA
Decay time constant (ms)
n.s.
C
I NMDA
100 ms
WT
PirB-/-
+ 40 mV
fast τ1
slow τ2
n.s.
Decay time constant (ms)
% contribution to the total I NMDA
D
0 20 50
150
300 ms
-70 mV
normalized
to 1st EPSC
WT
PirB-/-
100 ms
Normalized EPSC amplitude
Time (ms)
E
22 mV
200 ms
-70 mV
2 µM NBQX, 50 µM APV, 10 µM SR95531
Firing rate (Hz)
Vh = -70 mV
PiRB-/-
Current injection (pA)
F
fEPSP slope (mV/ms)
Stimulus Intensity (µA)
**
***
Fiber Volley (mV)

Figure S2

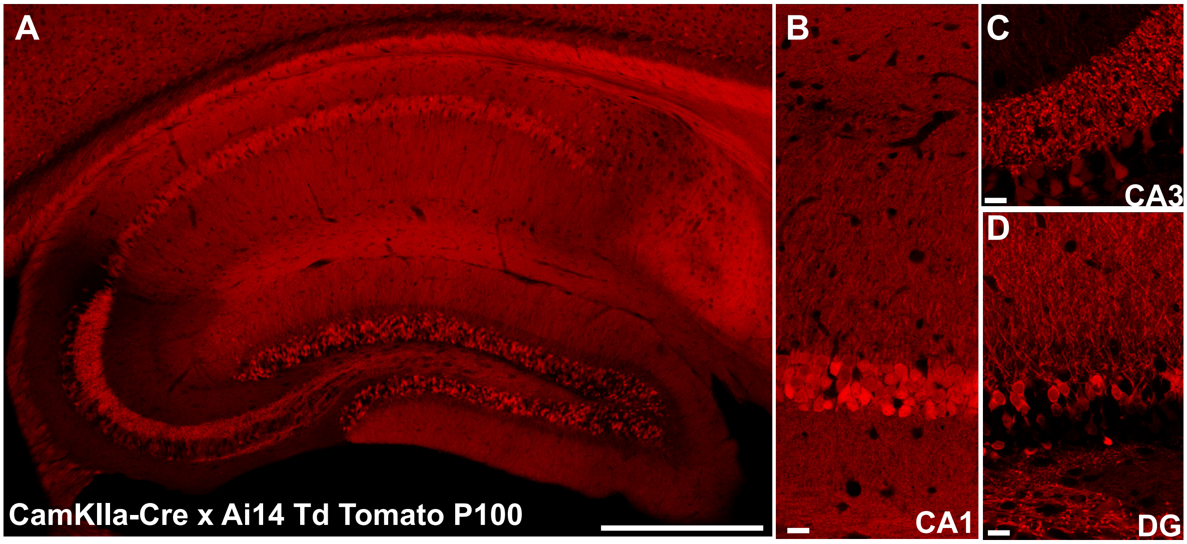

Figure S3

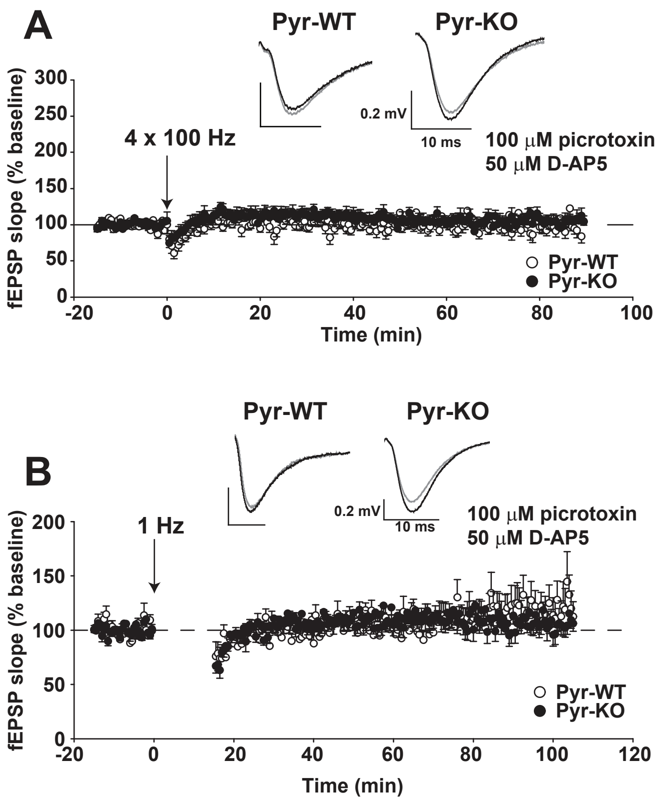
A
Pyr-WT
Pyr-KO
0.2 mV
10 ms
4 x 100 Hz
100 μM picrotoxin
50 μM D-AP5
fEPSP slope (% baseline)
Pyr-WT
Pyr-KO
Time (min)
B
Pyr-WT
Pyr-KO
0.2 mV
10 ms
1 Hz
100 μM picrotoxin
50 μM D-AP5
fEPSP slope (% baseline)
Pyr-WT
Pyr-KO
Time (min)

Figure S4

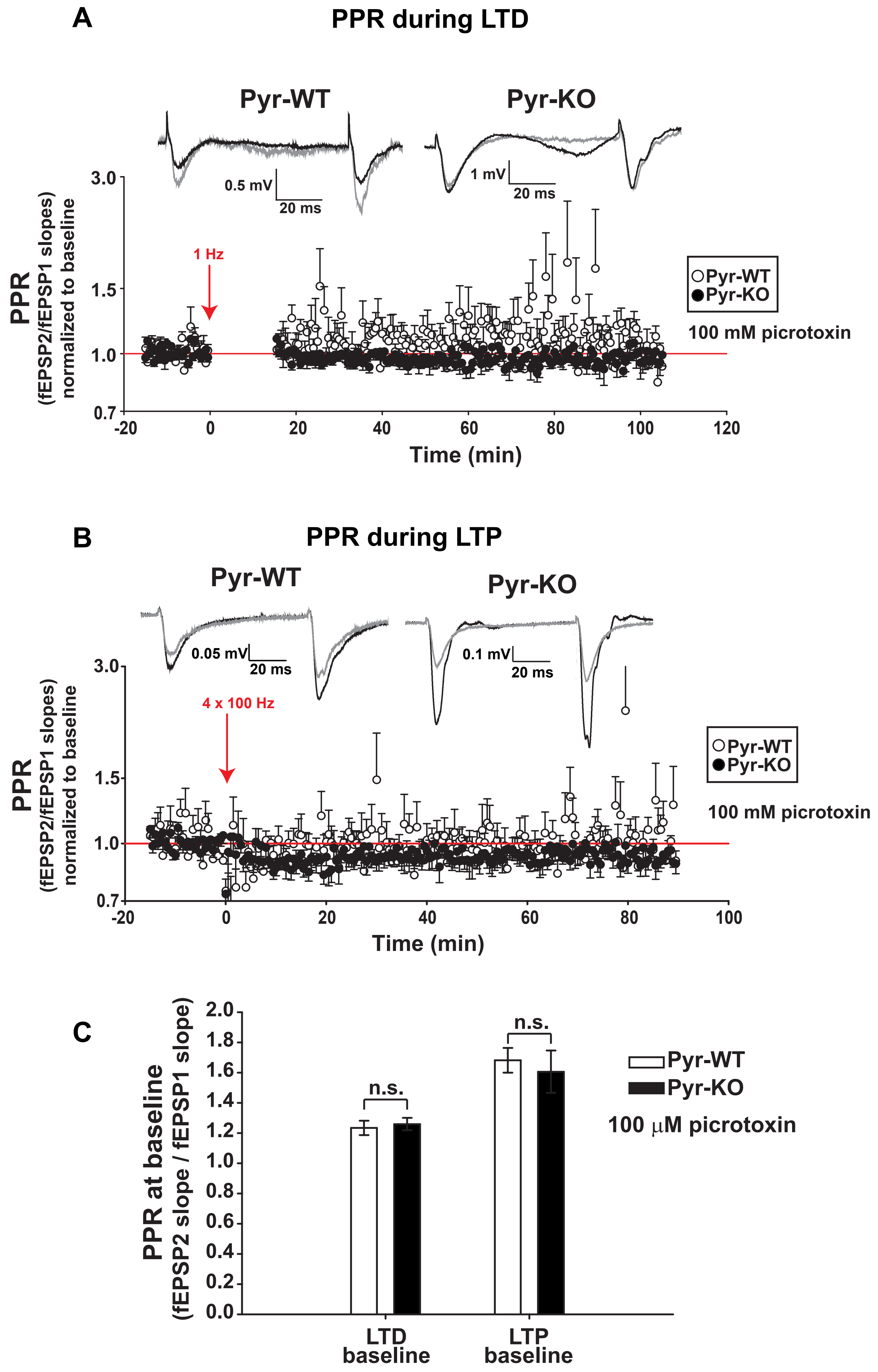
A
PPR during LTD
Pyr-WT
Pyr-KO
0.5 mV
20 ms
1 mV
20 ms
1 Hz
PPR
(fEPSP2/fEPSP1 slopes)
normalized to baseline
Time (min)
Pyr-WT
Pyr-KO
100 mM picrotoxin
B
PPR during LTP
Pyr-WT
Pyr-KO
0.05 mV
20 ms
0.1 mV
20 ms
4 x 100 Hz
PPR
(fEPSP2/fEPSP1 slopes)
normalized to baseline
Time (min)
Pyr-WT
Pyr-KO
100 mM picrotoxin
C
PPR at baseline
(fEPSP2 slope / fEPSP1 slope)
n.s.
n.s.
Pyr-WT
Pyr-KO
100 μM picrotoxin
LTD
baseline
LTP
baseline

Figure S5

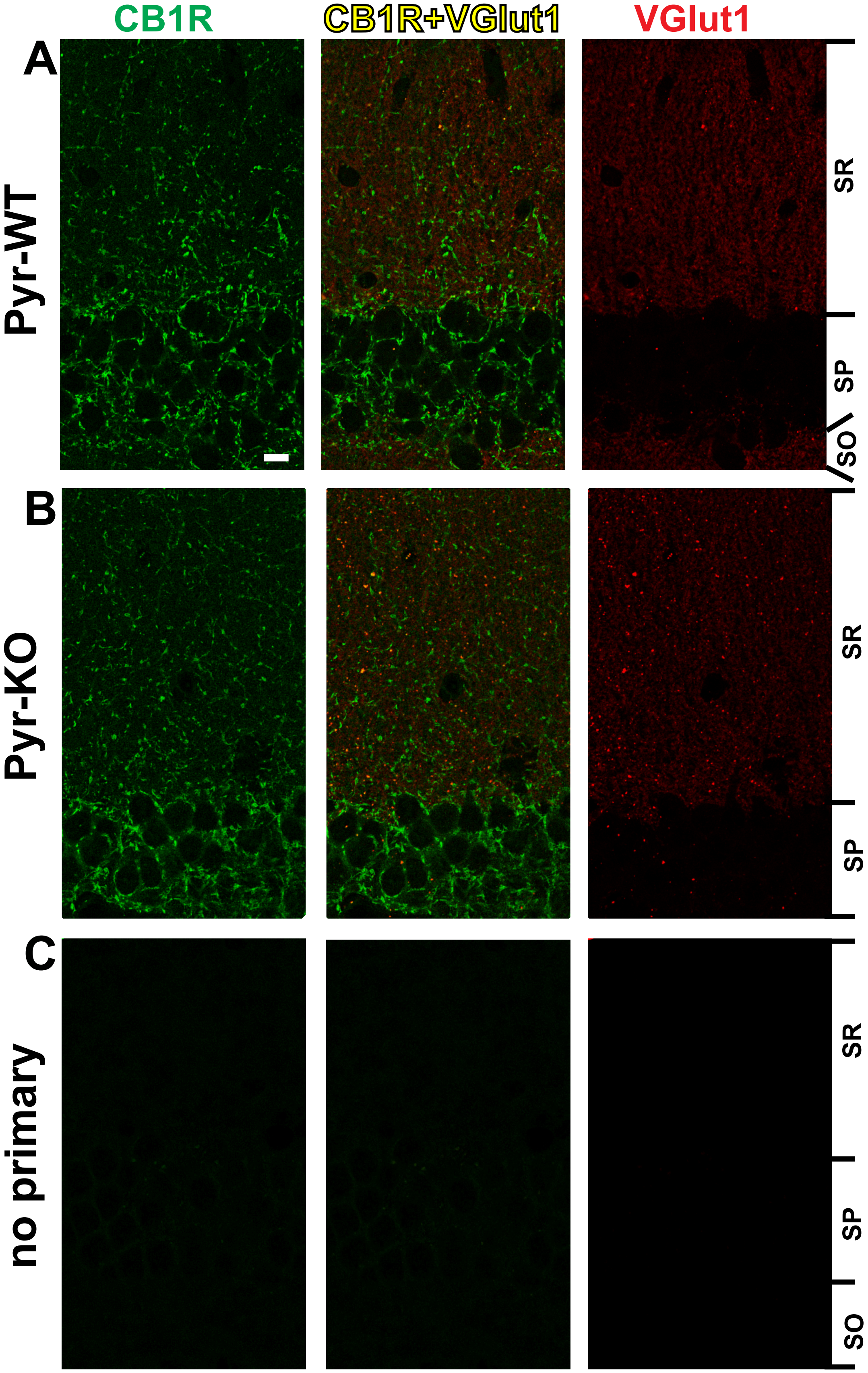
CB1R
CB1R+VGlut1
VGlut1
A
Pyr-WT
B
Pyr-KO
C
no primary
SR
SP
SO
SR
SP
SR
SP
SO

Figure S6

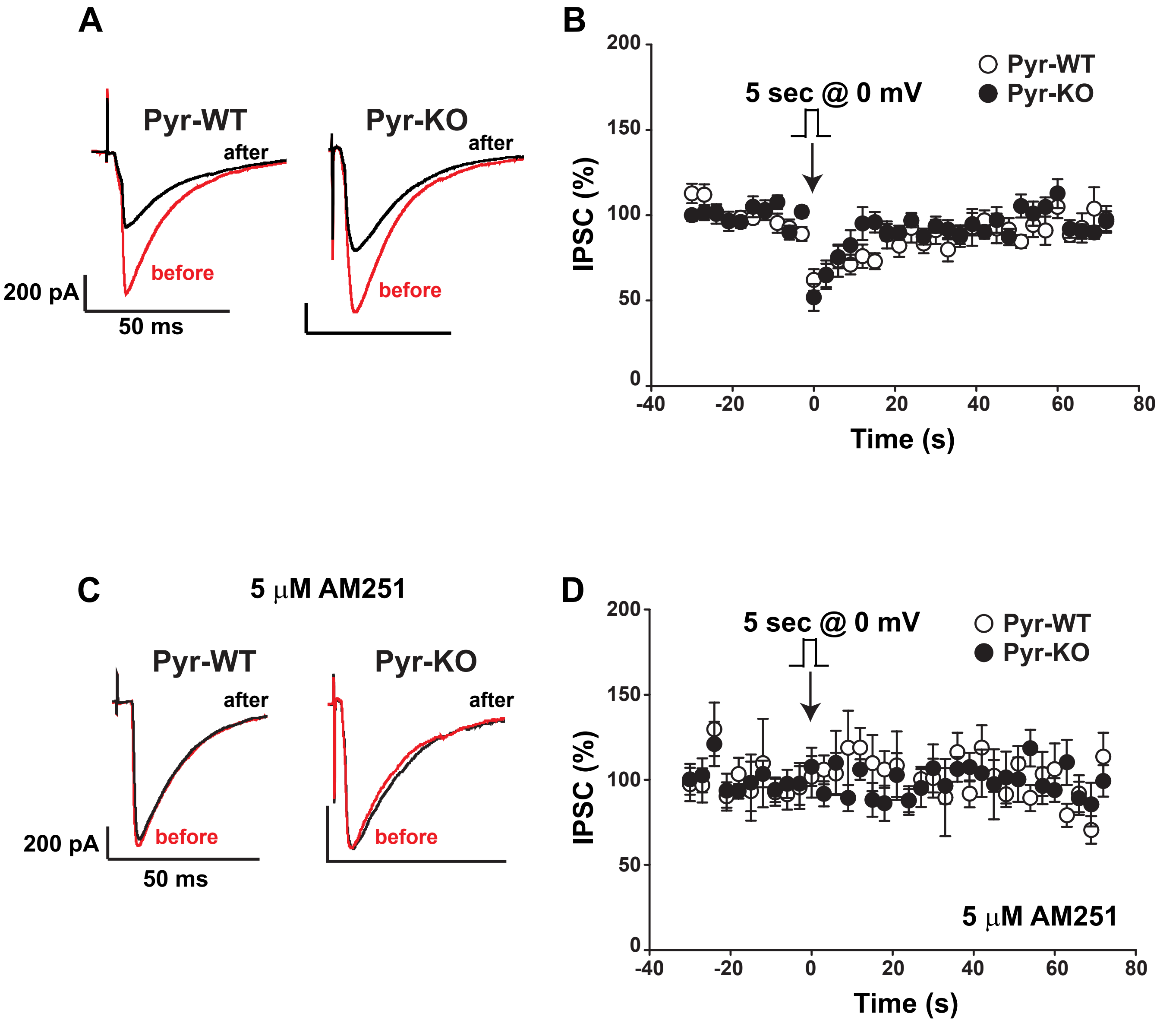
A
Pyr-WT
after
before
200 pA
50 ms
Pyr-KO
after
before
B
5 sec @ 0 mV
Pyr-WT
Pyr-KO
IPSC (%)
Time (s)
C
5 μM AM251
Pyr-WT
after
before
200 pA
50 ms
Pyr-KO
after
before
D
5 sec @ 0 mV
Pyr-WT
Pyr-KO
IPSC (%)
5 μM AM251
Time (s)

Figure S7

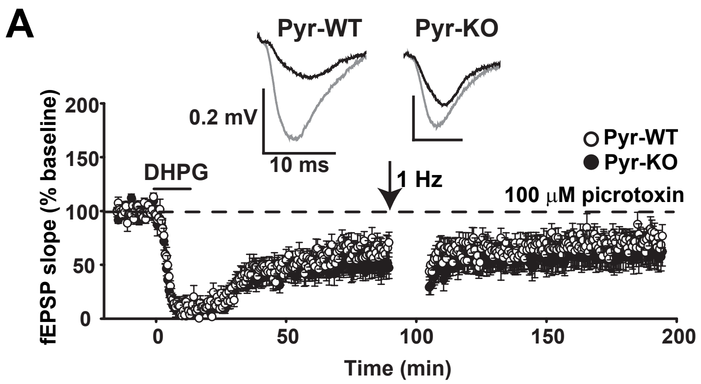
A
Pyr-WT
Pyr-KO
0.2 mV
10 ms
fEPSP slope (% baseline)
200
150
100
50
0
DHPG
1 Hz
Pyr-WT
Pyr-KO
100 μM picrotoxin
0
50
100
150
200
Time (min)
B
Pyr-WT
Pyr-KO
PPR
(fEPSP2 slope / fEPSP1 slope)
3.5
3.0
2.5
2.0
1.5
1.0
0.5
0.0
n.s.
n.s.
30-90 min
post DHPG
post 1 Hz

Figure S8

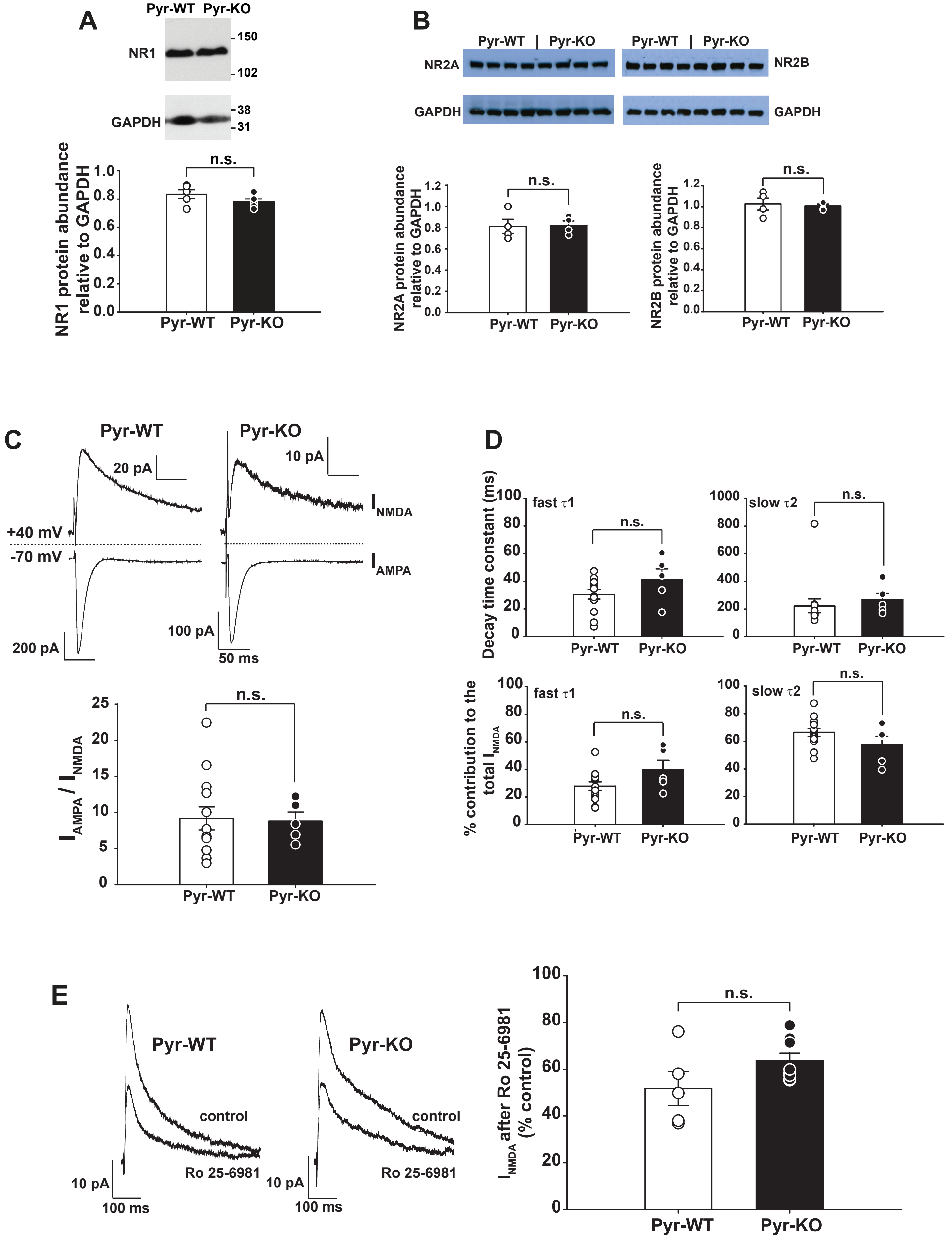
A
Pyr-WT Pyr-KO
NR1
GAPDH
150
102
38
31
NR1 protein abundance relative to GAPDH
n.s.
B
NR2A
NR2B
GAPDH
NR2A protein abundance relative to GAPDH
NR2B protein abundance relative to GAPDH
C
Pyr-WT
Pyr-KO
+40 mV
-70 mV
20 pA
10 pA
200 pA
100 pA
50 ms
I NMDA
I AMPA
I AMPA / I NMDA
D
Decay time constant (ms)
fast τ1
slow τ2
% contribution to the total I NMDA
E
control
Ro 25-6981
10 pA
100 ms
I NMDA after Ro 25-6981 (% control)

Figure S9

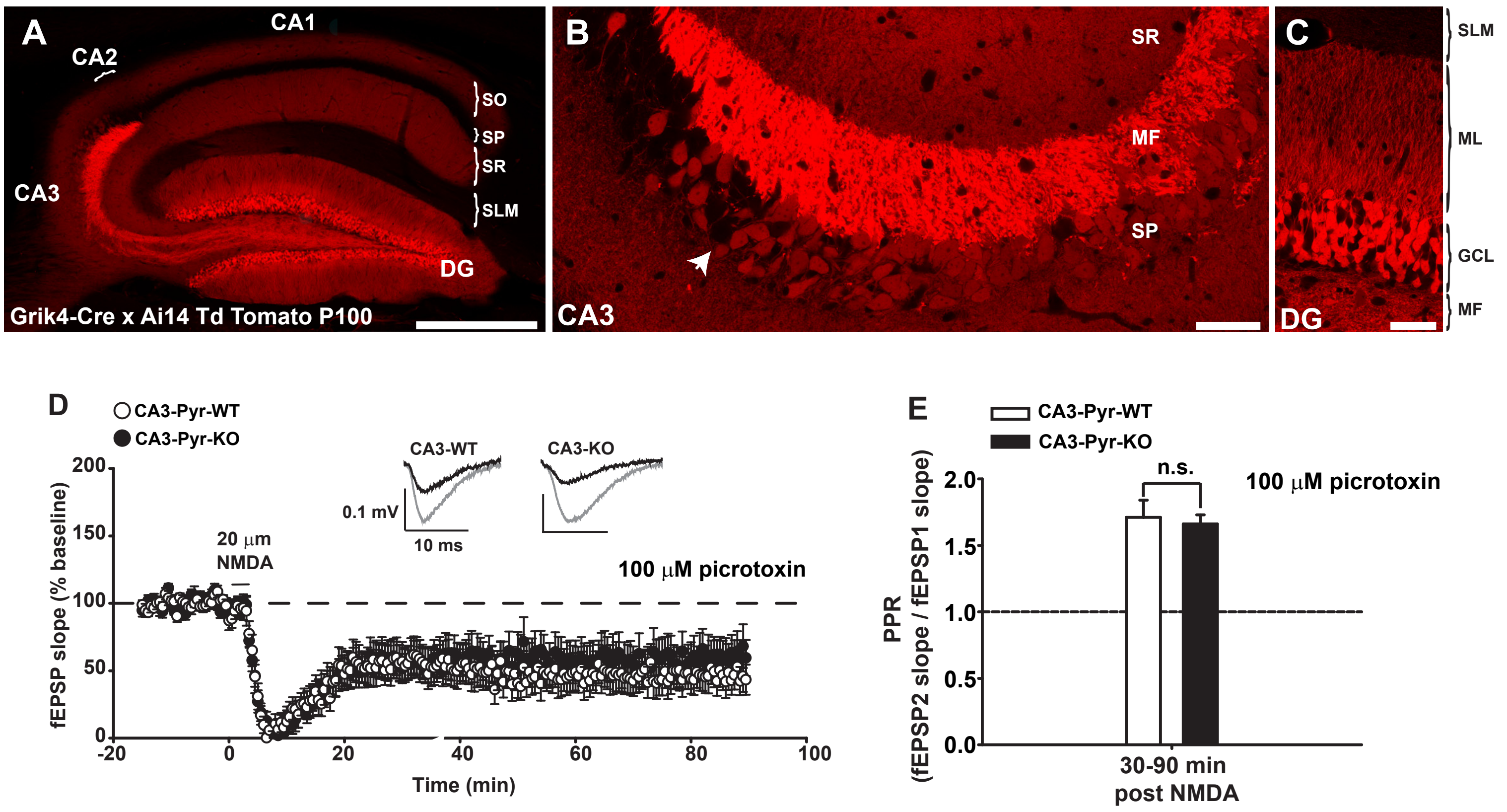
A
CA1
CA2
CA3
SO
SP
SR
SLM
DG
Grik4-Cre x Ai14 Td Tomato P100
B
SR
MF
SP
CA3
C
SLM
ML
GCL
MF
DG
D
CA3-Pyr-WT
CA3-Pyr-KO
CA3-WT
CA3-KO
0.1 mV
10 ms
20 µm NMDA
100 µM picrotoxin
fEPSP slope (% baseline)
Time (min)
E
CA3-Pyr-WT
CA3-Pyr-KO
n.s.
100 µM picrotoxin
PPR (fEPSP2 slope / fEPSP1 slope)
30-90 min post NMDA

Figure S10

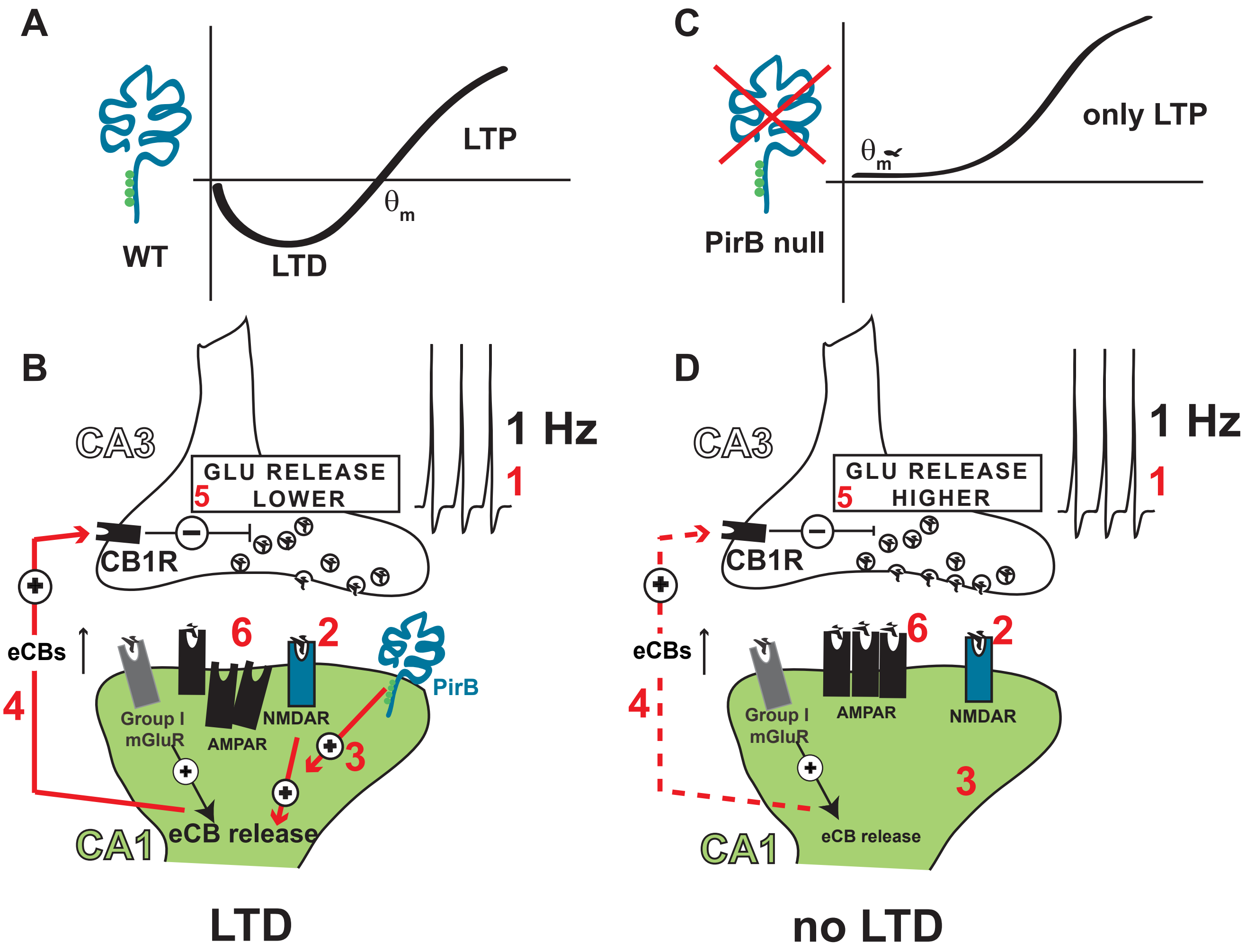
A
WT
LTP
LTD
θ_m
B
CA3
1 Hz
GLU RELEASE
5 LOWER
1
CB1R
eCBs
4
6
2
Group I
mGluR
AMPAR
NMDAR
PirB
3
CA1
eCB release
LTD
C
PirB null
θ_m
only LTP
D
CA3
1 Hz
GLU RELEASE
5 HIGHER
1
CB1R
eCBs
4
6
2
Group I
mGluR
AMPAR
NMDAR
3
CA1
eCB release
no LTD

Figure S11
